# Potential-Modulated Surface-Enhanced Raman Spectroscopy of Tolmetin at Gold Nanoparticle Film Functionalized Polarizable Liquid–Liquid Interfaces

**DOI:** 10.1021/acs.jpcc.4c00937

**Published:** 2024-05-04

**Authors:** Madjid Tarabet, Nataly Rey Muñoz, Micheál D. Scanlon, Grégoire Herzog, Manuel Dossot

**Affiliations:** † Université de Lorraine, CNRS, LCPME, F-54000 Nancy, France; ‡ The Bernal Institute and Department of Chemical Sciences, School of Natural Sciences, University of Limerick (UL), Limerick V94 T9PX, Ireland

## Abstract

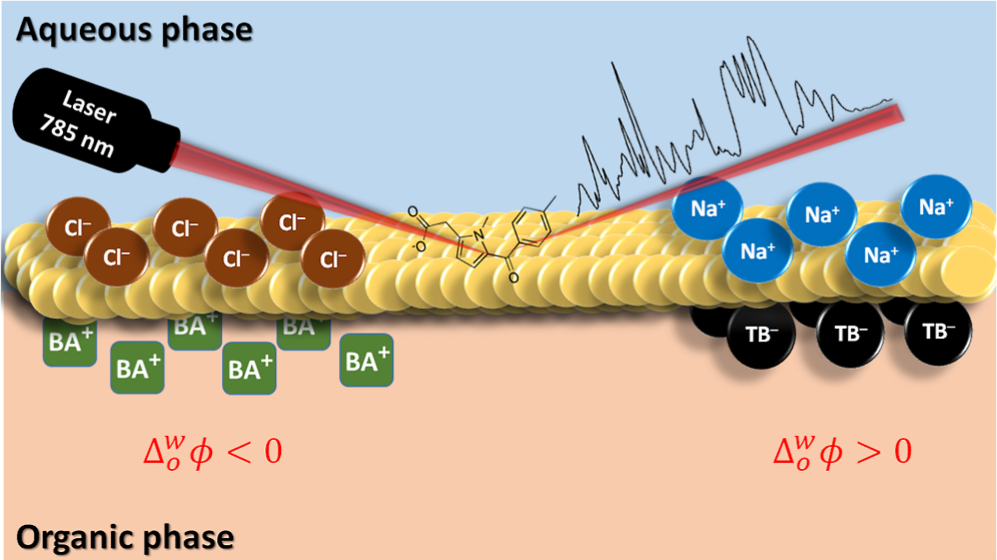

An aqueous colloidal suspension of gold nanoparticles (AuNPs) may be condensed into a thin fractal film at the polarizable liquid–liquid interface formed between two immiscible electrolyte solutions upon injection of millimolar concentrations of sodium chloride to the aqueous phase. By adjusting the interfacial polarization conditions (negative, intermediate, and positive open-circuit potentials), the morphology of the film is modified, resulting in unique surface plasmon properties of the film, which enable in situ surface-enhanced Raman spectroscopy (SERS). Intense SERS signals are observed at the polarizable liquid–liquid interface when micromolar concentrations of tolmetin, a nonsteroidal anti-inflammatory drug, are entrapped in the AuNP fractal film. The change in the signal intensity, averaged over multiple spectra, with respect to the concentration of tolmetin, depends on the polarization conditions and suggests the presence of chemical-induced damping effects on the surface plasmons of the gold film.

## Introduction

Surface-enhanced Raman spectroscopy (SERS) enables the strong enhancement of Raman scattering signals from molecules adsorbed on plasmonic nanostructures, as well as providing the vibrational fingerprint of a molecule and a high spatial resolution when coupled to confocal microscopy.^1−5^ Two broad strategies are typically employed to prepare SERS substrates that are generally, though not exclusively, metallic (gold or silver) in nature. The first approach is relatively low-cost and involves the aggregation of metallic nanostructures (nanoparticles, rods, spheres, stars, etc.) using salts, solvent evaporation, or centrifugation at either the solid–liquid or liquid–liquid interface.^6−9^ The films formed have predominately fractal morphologies and very high Raman enhancement factors (>10^8^ to 10^10^), though with a certain variability of reproducibility.^10−12^ The second approach is to lithographically form metallic nanostructured substrates with a highly controlled and reproducible organization, though at a higher fabrication cost and with comparatively lower Raman enhancement factors (typically of the order 10^5^).^13,14^

Plasmonically active, metallic nanostructure functionalized interfaces between two immiscible liquids (e.g., aqueous and organic phases) may be formed via reductive interfacial synthesis of a gold salt precursor,^15^ by self-assembly of metallic nanostructures at aqueous–organic interfaces,^16−23^ or by forming metallic films after stirring and sedimentation.^24^ These “plasmonic soft interfaces” provide unique advantages and a large parameter space (e.g., interparticle distance, degree of order, relative orientation, etc.) within which to create highly reproducible, scalable, and low-cost SERS substrates with high Raman enhancement factors.^25,26^ For example, the molecule(s) under analysis can be solubilized in either the aqueous or organic phase,^27^ and deleterious effects associated with drying solutions on solid SERS substrates that significantly impact the nature and reproducibility of the SERS signal (e.g., denaturation of biomolecules, uncontrolled reorganization of the analyte molecule at the film surface, uncontrolled aggregate formation, etc.) are circumvented.^27,28^ Critically, the parameter space available is significantly expanded in comparison to that using similar approaches at solid–liquid interfaces or using lithography. The chemical parameters of the two phases can be manipulated to enhance the adsorption of molecules onto the interfacial metallic films, e.g., the nature and concentration of the aqueous and organic electrolyte salts, aqueous pH, polarity of the organic solvent, and interfacial tension. Furthermore, for certain polarizable aqueous–organic interfaces formed using relatively polar organic solvents such as α,α,α-trifluorotoluene (TFT), the applied interfacial Galvani potential difference () can be manipulated by the interfacial distribution of salts or externally using a 4-electrode electrochemical cell.^29−33^

Herein, we demonstrate that the SERS signal of an analyte molecule at a gold nanoparticle (AuNP) film functionalized polarizable liquid–liquid interface can be modulated by the applied . Polarizing salts contain a hydrophobic cation and a hydrophilic anion, or vice versa, and upon their introduction to the organic phase, the hydrophilic cation/anion distributes between both phases and polarizes the liquid–liquid interface.^32^ In this work, polarizing salts that establish open-circuit potentials (OCPs) at  values at the positive and negative extremes of the polarizable potential window (PPW) are investigated, and their influence on the SERS signal compared to that under OCP conditions established by a salt containing both a hydrophobic cation and an anion (neither of which partition out of the organic phase within the PPW). Polarization of the liquid–liquid interface influences the ion distributions in the electrical double-layers on both sides of the interface,^34^ which in turn influences the morphology of the interfacial AuNP films formed and, thus, the nature of the SERS signal recorded. Additionally, polarization of the liquid–liquid interface can influence the SERS signal by enriching or depleting the concentration of the analyte (if it is ionic in nature) in the interfacial region or lead to the formation of interfacial ion pairs between the ionic analyte and oppositely charged electrolyte ion in the adjacent phase, e.g., the formation of an ion pair between an anionic aqueous analyte and the organic electrolyte cation.

The influence of the applied  on the SERS signal of tolmetin, a nonsteroidal anti-inflammatory drug (NSAID), at a AuNP film functionalized polarizable liquid–liquid interface is investigated. The release of tolmetin into environmental waters poses numerous problems in terms of public health and for wild flora and fauna.^35^ Costly methods based on coupled chromatographic techniques are currently used to detect tolmetin in tap or environmental water.^36^ SERS will be demonstrated as a viable alternative approach to detect tolmetin at low concentrations in water samples, typically below 1 μM and at least qualitatively, to create low-cost sensors as an early warning system using an approach involving the aggregation of metallic nanoparticles in an electrochemically controlled ionic environment at a polarizable liquid–liquid interface.

## Experimental Methods

### Chemical and Reagents

All chemicals were used as received, and all aqueous solutions were prepared from ultrapure water (18.2 MΩ·cm, Purelab Option-Q from Elga). The glassware used was cleaned by sequential immersion in (i) an oxidizing solution of 0.1 M potassium permanganate (KMnO_4_, 99.5%, Prolabo) and 90 mM sulfuric acid (H_2_SO_4_, Sigma-Aldrich) and (ii) a solution of H_2_SO_4_ and hydrogen peroxide (H_2_O_2_, Sigma-Aldrich) (1:3 v/v). The glassware was then rinsed with boiling water and acetone. AuNPs were synthesized using gold chloride trihydrate (HAuCl_4_, 99.9%, Sigma-Aldrich) and sodium citrate (ACS, ISO, Reag PhEur reagents, Merck).

The formation of the interfacial AuNP film requires sodium chloride (NaCl, 99.9%, Fluka). The analyte detected by SERS was tolmetin sodium dihydrate (98%+, Alfa-Aesar). The organic electrolytes used were bis(triphenylphosphoranylidene)ammonium chloride (BACl, 97%, Sigma-Aldrich) and lithium tetrakis(pentafluorophenyl)borate diethyl etherate ([LiTB, Boulder Scientific company). Bis(triphenylphosphoranylidene)ammonium tetrakis(pentafluorophenyl)borate (BATB) was synthesized from equimolar solutions of BACl and LiTB in a mixture (2:1 v/v) of water and methanol. The resulting precipitate was filtered, washed, dissolved in acetone, and recrystallized. The crystals obtained were washed twice with a mixture (1:1 v/v) of water and acetone. Glass silanization was carried out with trimethylchlorosilane (98%, Sigma-Aldrich). The organic solvents used were TFT (99%+, Sigma-Aldrich) and methanol (Carlo Erba).

### AuNP Synthesis

The Turkevich synthesis method with modified citrate concentrations was used to synthesize AuNPs. The main steps of the synthesis, illustrated in Figure S1, Supporting Information, included first the heating (up to the boiling point) of 50 mL of a solution of 1 mM HAuCl_4_ solution under stirring and then the addition of 5 mL of a 33 mM sodium citrate solution (as a reducing agent). The solution progressively changed color from pale yellow to colorless, then to violet, and finally to ruby red. This indicated the reduction of Au^3+^ ions to Au^0^. At this point, the reaction was left to cool down at room temperature. The colloidal suspension was stored in glass vials at 4 °C.

### Interfacial AuNP Film Assembly

An essential first step toward interfacial AuNP assembly is to silanize the section of the cylindrical glass cell (inner diameter of 27 mm) in contact with the organic phase by injecting 5 mL of a solution containing 20 μL of trimethylchlorosilane mixed into 20 mL of TFT. After 20 s of the reaction of trimethylchlorosilane with the glass walls, the silanizing solution was carefully removed, and the glassware was washed with water to stop the reaction. Silanization renders the lower section of the cylindrical vials hydrophobic and enables as flat a liquid–liquid interface as is possible to be achieved.

For the AuNP assembly, 5 mL of the denser organic phase, composed of TFT solvent and either 1 mM BACl or 2.5 mM LiTB or BATB as the organic salt, was poured into the vessel. Next, the AuNP (and if applicable tolmetin)-containing aqueous phase was added dropwise on the top of the organic phase. The AuNP film assembly was induced by the addition of an aliquot of 100 μL of a 5 M NaCl solution to the aqueous phase to reach a final concentration of 100 mM. The increase in ionic strength triggered the aggregation of the AuNPs, and the aqueous solution changed color from red to dark violet. The biphasic system was allowed to reach equilibrium for 5 h before performing any measurements.

Extensive details of the various characterization methods employed for both the aqueous suspension of colloidal AuNPs and the interfacial AuNP film formed are provided in the Supplementary Experimental Methods section of the Supporting Information. The characterization methods include UV–vis absorption spectroscopy, in situ visible absorption spectroscopy at the polarized liquid–liquid interface, dynamic light scattering (DLS), transmission electron microscopy (TEM), scanning electron microscopy (SEM), and Raman spectroscopy.

## Results and Discussion

### Synthesis and Characterization of the AuNPs

The Turkevich method^37^ with modified citrate concentrations was used to synthesize AuNPs, as summarized in Figure S1, Supporting Information. The AuNPs were characterized by UV–vis absorption spectroscopy, TEM, DLS, and zeta (ξ) potential measurements. Both the average particle diameter (*d*) and average number of AuNPs per mL of suspension (*N*) were consistent between synthesis batches (Figure S2, Supporting Information). The average particle diameter was consistent across the different experimental methods employed including UV–vis absorption spectroscopy (22.0 ± 2.5 nm), TEM (22 ± 5 nm), and DLS (27 ± 4 nm), see Figures S3–S5A, Supporting Information. The average number of AuNPs was *N* = (1.48 ± 0.09) × 10^11^ per mL of suspension. The average ξ-potential of the AuNPs was −44 ± 4 mV (Figure S5B, Supporting Information), confirming the capping of the surface of the AuNPs with negatively charged citrate molecules.

### Inducing AuNP Film Assembly at the Liquid–Liquid Interface

**Scheme 1:**
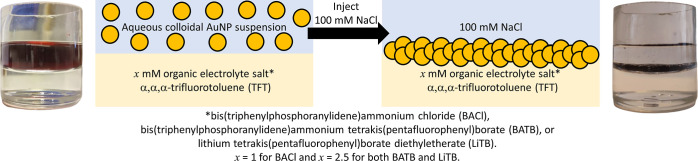
Optical Images of the Stable Aqueous Colloidal AuNP Suspension in Contact with the Organic Phase of 2.5 mM Organic Electrolyte Salt in TFT Solvent and an Interfacial AuNP Film Formed at the Aqueous–TFT Interface Upon Injection of 100 mM NaCl to the Aqueous Colloidal AuNP Suspension The organic electrolyte salts investigated were BACl, BATB, and LiTB, each of which polarize the aqueous–TFT interface at a different OCP.

To investigate the formation of an interfacial AuNP film, a biphasic system was used with a lower-density aqueous phase containing the colloidal AuNP suspension and a higher density organic phase containing the organic electrolyte salt BATB at a concentration of 2.5 mM in TFT solvent, see Scheme 1. The chemical structure of BATB is shown in Figure S6, Supporting Information. The AuNP suspension was stable upon contact of the immiscible aqueous and organic phases to create the liquid–liquid interface. Thus, interfacial AuNP film assembly requires the destabilization of the bulk AuNP suspension to induce aggregation, followed by the sedimentation of these aggregates to form a AuNP film at the aqueous–TFT interface.^38,39^ A simple method to induce AuNP aggregation is to introduce NaCl (at a final concentration of 100 mM) to the aqueous phase. The Na^+^ ions screen the negative charges of the citrate-capped AuNPs, reducing the repulsive forces between individual AuNPs and thereby promoting their aggregation with time and deposition at the interface.

**Figure 1:**
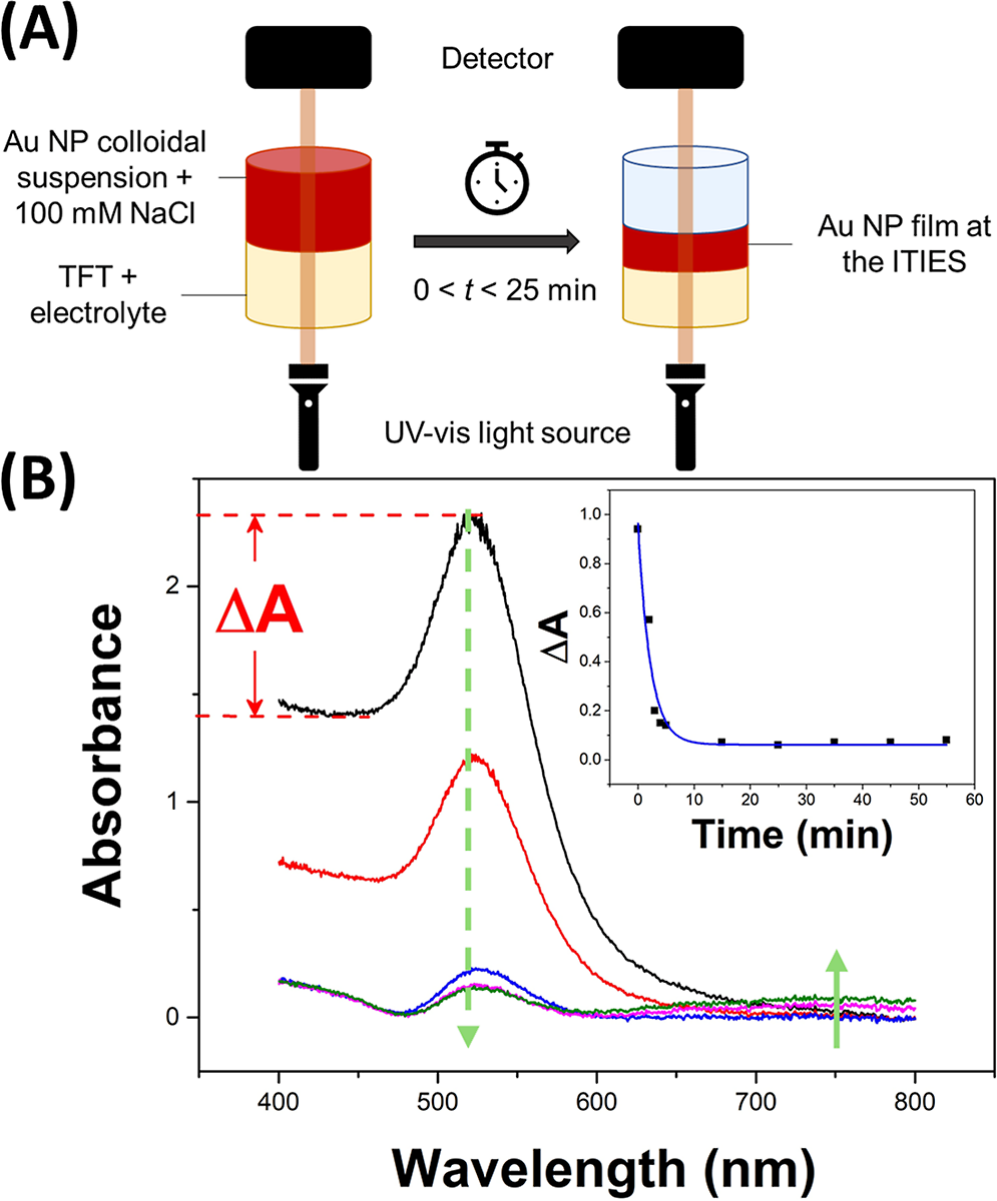
(A) Schematic of the experimental setup for the in situ measurement of UV–vis absorption spectra in the transmission mode to follow the induction of AuNP aggregation by the addition of NaCl and the associated interfacial AuNP film assembly. (B) Effect of the addition of NaCl to the AuNP colloidal suspension on the UV–vis absorption spectra at *t* = 0, 2, 5, 10, and 25 min. The green dashed line arrow represents the decrease of the absorbance at the wavelength of the SPR band of the colloidal AuNPs (λ_SPR_). The small green arrow indicates the absorbance increase due to the formation of the plasmonic band of the AuNP film growing at the interface between two immiscible electrolyte solutions (ITIES). Inset: Δ*A* (= *A*_SPR_ – *A*_0_) as a function of time. The blue line is a fit by a mono exponential decay, see the text.

As discussed in Figure S1, Supporting Information, the AuNPs were synthesized through a modified Turkevich method that was optimized to facilitate fast aggregation of the AuNPs upon NaCl addition. In this regard, the rate of formation of the interfacial AuNP film was investigated in situ by measuring the UV–vis absorption spectra in the transmission mode over time with a UV–vis light source and a spectrophotometer arranged perpendicular to the aqueous–TFT interface (Figure 1A). Prior to adding NaCl (*t* = 0), the spectrum of the colloidal AuNP suspension was stable, with a surface plasmon resonance (SPR) band centered at λ_SPR_ = 523 ± 0.5 nm (black spectra, Figure 1B, green dashed arrow). However, at *t* = 2 min after NaCl addition (red spectra, Figure 1B), the absorbance decreased by 50%, suggesting the formation of AuNP aggregates and their accumulation at the aqueous–TFT interface. With time, light scattering also increased due to the presence of an interfacial AuNP film (green arrow in Figure 1B), and consequently, the absorbances in Figure 1B were followed over time by measuring Δ*A*, the difference between *A*_SPR_ and *A*_0_ (the absorbance values at λ_SPR_ and λ_0_, and the local minimum wavelength at 450 nm in Figure 1B). Figure 1B shows how Δ*A* changed over time. The data points were fitted by a mono exponential decay (blue line in Figure 1B, inset) following the corresponding equation: . The fitted parameter values were as follows: *A*_0_ = 0.061 ± 0.004 (the residual absorbance due to free AuNP particles not yet being aggregated or incorporated into the film), *A*_1_ = 0.882 (the initial absorbance of the plasmonic band of the AuNP, calculated as indicated in Figure 1B), *t*_0_ = 0.05 min (a small time shift due to the introduction of the NaCl spike in the aqueous phase), and *t*_1_ = 2.21 ± 0.38 min (the exponential decay time). The *R*-square value was 0.9328 and the reduced Chi-square value was 0.0057. Parameter *t*_1_ is the characteristic time for the AuNPs to form aggregates in the aqueous phase, leading to a strong decrease of the plasmonic band at 523 nm of the non-aggregated AuNPs. This is not the time required to form the entire film since aggregates will further sediment to the interface between two immiscible electrolyte solutions (ITIES) and keep on growing to form the final fractal film in several hours. Nevertheless, interfacial AuNP film assembly at the aqueous–TFT interface was shown to be a quick process, with Δ*A* dropping quickly after the addition of NaCl and reaching a low steady-state value (0.06 absorbance unit) after only 15 min. DLS experiments as a function of NaCl concentration revealed that the AuNP aggregate sizes increased with NaCl concentrations ≥50 mM in the aqueous suspension (Figure S7, Supporting Information).

### Influence of Polarization of the Aqueous–TFT Interface on the Morphology of the Interfacial AuNP Film

Polarization of the aqueous TFT interface was modulated chemically to establish OCPs at  values at the positive and negative extremes of the PPW, as well as at an intermediate  value approximately midway between both extremes. As shown in Figure S8, Supporting Information, OCPs of −0.439, −0.288, and +0.691 V on the Galvani scale were obtained when 1 mM BACl, 2.5 mM BATB, or 2.5 mM LiTB electrolyte salts, respectively, were dissolved in the TFT solvent. The OCP measurements were obtained using an aqueous phase of 100 mM NaCl, i.e., in the absence of the colloidal AuNPs.

**Figure 2:**
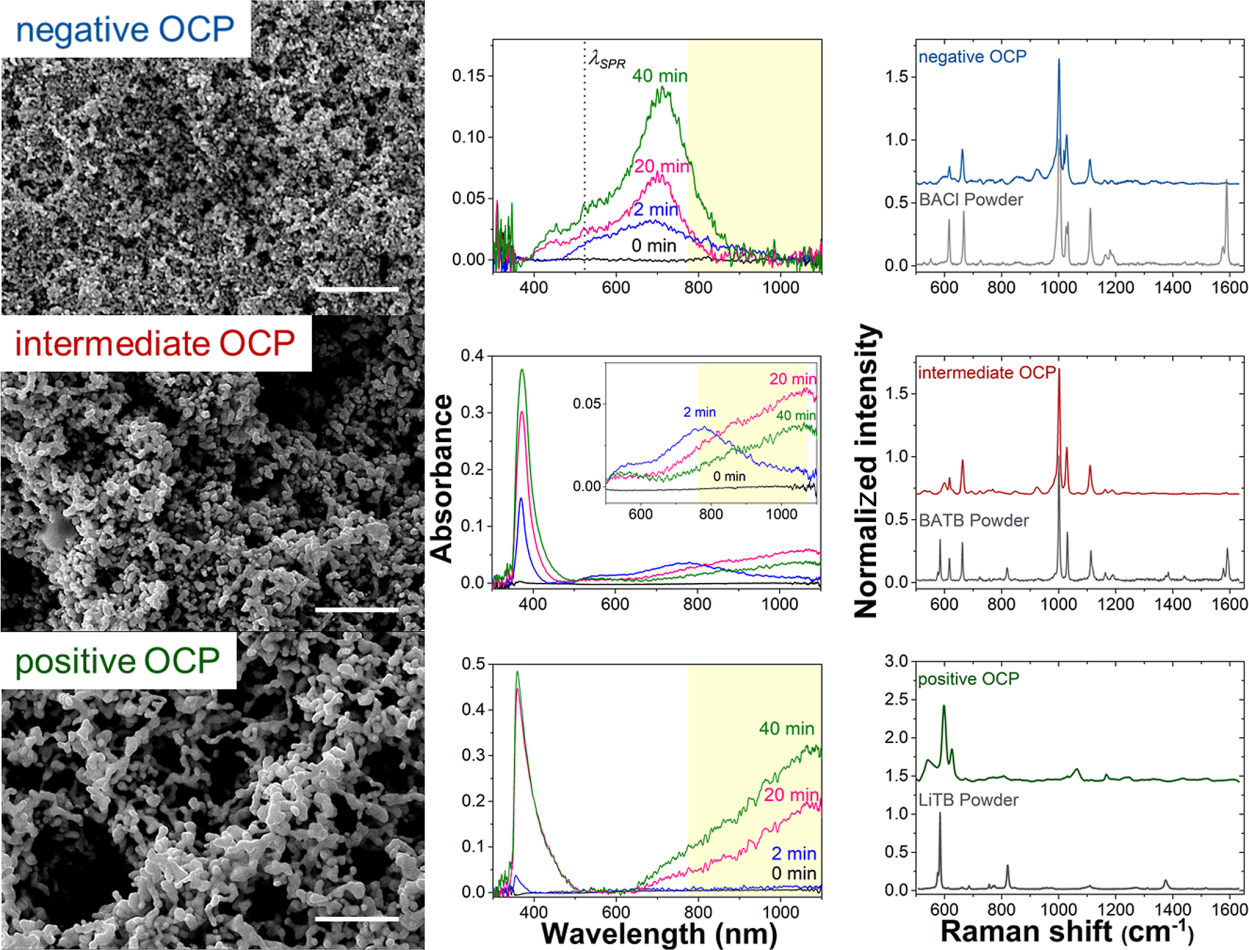
Influence of polarization of the aqueous–TFT interface on the morphology of the interfacial AuNP films and the in situ UV–vis absorption and SERS spectra obtained in the presence of interfacial AuNP films formed at each OCP condition. The aqueous–TFT interface was polarized at a negative OCP on the Galvani scale of −0.439 V using the BACl electrolyte (top row), an intermediate OCP of −0.288 V using the BATB electrolyte (middle row), and a positive OCP of +0.691 V using the LiTB electrolyte (bottom row). Interfacial AuNP film assembly was induced by adding 100 mM NaCl to the aqueous phase containing the colloidal AuNPs and the concentration of the organic electrolyte was 1 mM for BACl and 2.5 mM for BATB and LiTB, respectively. (Left column) SEM images of the morphologies of AuNP films formed at an aqueous–TFT interface as a function of the interfacial polarization. The scale bar represents 1 μm. (Middle column) UV–vis spectroscopy in the TIR mode to monitor the UV–vis absorption bands arising from the plasmonic properties of AuNP films as they assembled with time (at *t* = 0, 2, 20, and 40 min). The yellow area indicates the Raman shift range to allow comparison of the SPR bands of the different interfacial AuNP films formed. The dashed line in the top panel represents the wavelength of the SPR band of the colloidal AuNPs (λ_SPR_). The inset of the middle panel is a magnification of the plasmonic band range. (Right column) Influence of the polarization of the aqueous–TFT interface on the SERS spectra obtained at interfacial AuNP films. The spectra in gray are the reference spectra of the organic electrolyte salts used to set each OCP condition in the powder form.

The influence of the OCP on the AuNP film morphology was analyzed by SEM. Interfacial AuNP films were formed at each OCP, carefully transferred from the aqueous–TFT interface, and collected on a TEM grid for SEM analysis. Distinct morphologies of the AuNP aggregates comprising each of the AuNP films were observed, as shown in Figure 2 (left column). At the negative OCP set using the BACl electrolyte, the film consisted of a dense packing of spherical AuNP aggregates with an average size below 200 nm. At the positive OCP set using the LiTB electrolyte, the AuNP aggregates formed a vermicular pattern resembling a coral reef structure, with continuous (but fractal) links between gold nanostructures. At the intermediate OCP set using the BATB electrolyte, the morphology was also intermediate between the two extremes, consisting of vermicular fractal structures but with a smaller average size than with the LiTB electrolyte. Further SEM images of each film are shown at a lower magnification in Figure S9, Supporting Information, to highlight the homogeneity of each film’s morphology. SEM analysis was also performed on AuNP films obtained in the presence of 1 mM tolmetin in the aqueous phase, the analyte to be investigated using SERS vide infra (Figure S10, Supporting Information). No discernible morphological difference with the films obtained in the absence of tolmetin were found, demonstrating that the tolmetin molecule does not influence the morphology of the interfacial AuNP films formed at each OCP investigated.

**Scheme 2:**
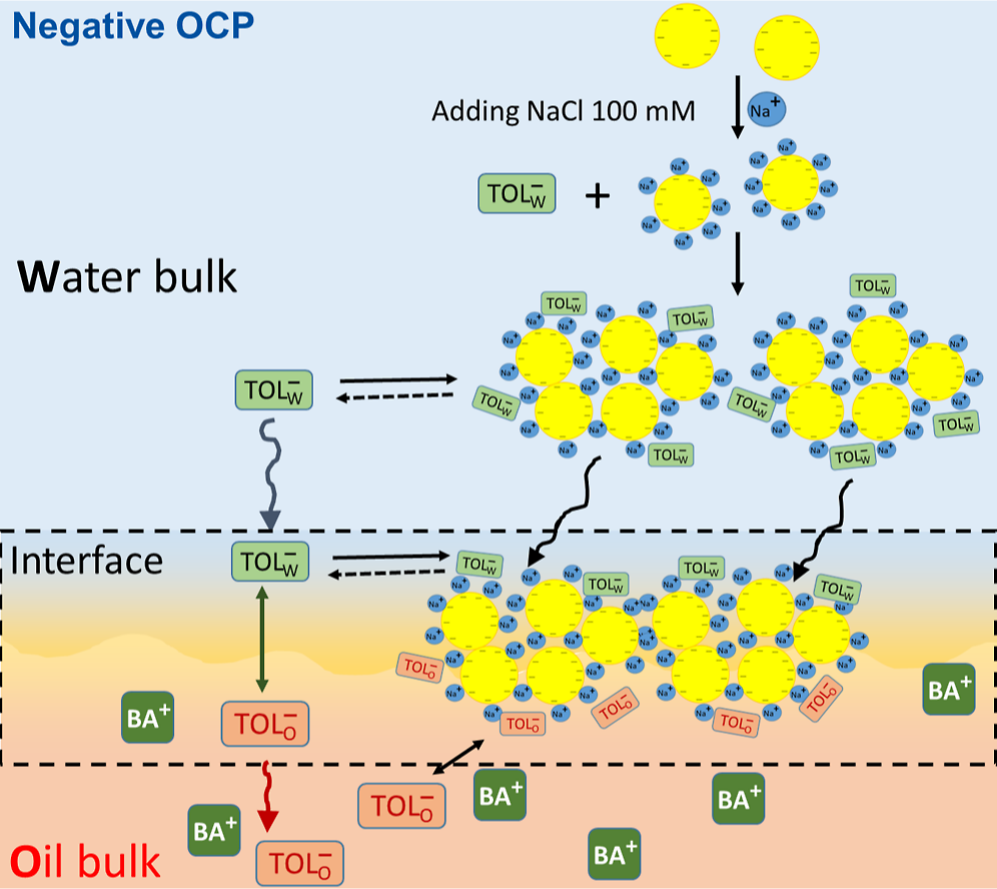
Upon the Addition of a High Concentration of NaCl to the Bulk Aqueous Phase, Na^+^ Cations in Solution Neutralized Some of the Negative Charge on the Citrate-Stabilized AuNPs This facilitated AuNP cluster formation and simultaneous adsorption of anionic tolmetin molecules on the AuNP clusters and ultimately within the interfacial AuNP film. This process occurred independent of the OCP.

The fractal dimensions of the AuNP films obtained in the absence and presence of 1 mM tolmetin were calculated for the three polarization values (see Figures S11 and S12, Supporting Information). In the absence of tolmetin, the fractal dimensions of the films were 1.86, 1.80, and 1.76 for the negative, intermediate, and positive OCP, respectively. An average fractal dimension of 1.8 is compatible with the diffusion-limited cluster aggregation (DLCA) model.^10^ At a high ionic strength (>75 mM), AuNPs capped by citrate molecules have been reported to aggregate, forming clusters, following this mechanism, while at lower ionic strength, the mechanism is reaction-limited instead of diffusion-limited.^40^ Herein, the 100 mM NaCl aqueous electrolyte imposed a high ionic strength, leading to rapid screening of the repulsive electrostatic interactions between negatively charged AuNPs and an ensuing diffusion-limited rapid aggregation process. The presence of tolmetin in the aqueous phase does not significantly impact the AuNP film morphology as the fractal dimension remained close to the ones obtained in the absence of tolmetin (see Section S3.3, Supporting Information). The fact that the fractal dimensions of the AuNP films are compatible with the DLCA model indicates that there is a first step in the film formation that involved the aggregation of AuNPs in small clusters. During this first step, if tolmetin molecules are present in the aqueous phase, they can strongly interact with the surface of these clusters. The latter will migrate to the interface, due to both sedimentation and interaction with the electric potential of the interface. The aggregated AuNPs therefore act like a trap for tolmetin molecules and favor its concentration at the interface. This mechanism in the aqueous phase is pictorially described in Scheme 2.

The coral reef structures observed at intermediate and positive OCPs indicate that some AuNPs have fused dense aggregates together, locally forming quasi-1D nanowires but keeping self-similarity and the fractal morphology. He et al. obtained a similar coral reef structure after aggregating citrate-stabilized AuNPs using cetyltrimethylammonium bromide (CTAB) solutions.^41^ These coral reef structures require the fusion of AuNPs with clusters, a process that requires the attractive van der Waals forces to counterbalance the repulsive electrostatic forces. The latter are partially screened by the high aqueous ionic strength imposed by CTAB, in the case of He et al., and NaCl herein. The distinct physicochemical conditions at a polarized liquid–liquid interface, compared to the bulk aqueous phase, may also favor the formation of this coral reef structures. For example, the positive OCP set by the LiTB electrolyte enhances the concentration of Na^+^ cations in the electrical double layer on the aqueous side of the liquid–liquid interface by an order of magnitude at least.^34^ This significant local increase in the aqueous cation concentration may diminish the distance between both AuNPs and their aggregates by further neutralizing the anionic electrostatic repulsive forces and, thereby, favor the fusion of AuNPs with existing aggregates present at the liquid–liquid interface. Conversely, the negative OCP set by the BACl electrolyte enhances the concentration of Cl^–^ anions in the electrical double layer on the aqueous side of the liquid–liquid interface, potentially increasing the inter-AuNP distances and, thereby, inhibiting the fusion of AuNPs with existing aggregates.

Based on this SEM analysis, due to the emergence of the coral reef structures at the intermediate OCP and a subsequent increase in the dimensions of these fractal structures at the positive OCP, an inference can be made that the gold surface area available for tolmetin to adsorb in the interfacial AuNP film decreases going from negative to intermediate to positive OCP values, respectively.

### In Situ UV–Vis Absorption and SERS Spectra in the Presence of Interfacial AuNP Films Formed at Different Polarizations of the Aqueous–TFT Interface

The technique of UV–vis spectroscopy in the total internal reflection (TIR) mode at a liquid–liquid interface can probe the nature of molecular or nanoparticulate species either adsorbed, self-assembled, or electrosynthesized at a polarized or unpolarized liquid–liquid interface. For example, using this in situ spectroscopic technique, interfacial porphyrin aggregation and cytochrome *c* denaturation have been explored.^42−44^ Herein, UV–vis spectroscopy in the TIR mode was used to monitor the UV–vis absorption bands arising from the plasmonic properties of AuNP films as they assembled over a 20 min period upon the addition of 100 mM NaCl to the aqueous phase (Figure 2 (middle column)). Using this approach, the influence of the changes of the morphology of the AuNP films as a function of the interfacial polarization on the evolution of the UV–vis spectra in the presence of the aqueous and organic background electrolyte ions only was investigated.

At the negative OCP set using the BACl electrolyte, a broad plasmonic band appeared with a peak maximum at ca. 700 nm after 2 min and increased in absorbance with time. Due to the aggregation of the AuNPs within the interfacial AuNP film, the latter’s plasmonic band was notably red-shifted compared to that of the individual AuNPs in the initial aqueous suspension (λ_SPR_ of 523 nm) and quite similar to those observed from salt-induced aggregation of gold colloids.^39,40^

At the intermediate OCP set using the BATB electrolyte, the interfacial AuNP film plasmonic band again appeared after 2 min, with a red shift of the peak maximum of ca. 769 nm compared to that at the negative OCP. The plasmonic band strongly shifted to the near-infrared with time, suggesting continued changes to the morphology of the interfacial AuNP film with time due to aggregate–aggregate coupling. The latter agrees with the morphology of the AuNP films observed by SEM, showing fused dense AuNP aggregates together that locally form quasi-1D nanowires. The small drift of the interface in the *z*-direction may explain the small decrease in absorbance observed at 40 min, compared to the spectrum observed at 20 min. A minor absorption band with a maximum close to 550 nm was also visible and may correspond to dimers or trimers of AuNPs.^45,46^ A significant absorption band with a peak maximum at 373 nm was attributed to the electronic absorption of the TB^–^ anion. The increase in magnitude of this absorbance band with time indicates that the chemical equilibria between the aqueous and organic electrolyte solutions and the interfacial AuNP film may require more time than the formation of the film itself (a process that takes <15 min, as shown in Figure 1B).

At the positive OCP set using the LiTB electrolyte, a very broad plasmonic band appeared after 20 min in the near-infrared part of the spectrum. This is typical for AuNP films consisting of either large aggregates (as observed herein by SEM for the interfacial AuNP films formed at a positive OCP)^47^ or nanoparticles of a particular morphology, such as gold nanotriangles.^48^ The tail of the absorption band of the free TB^–^ anion is visible at 360 nm (blue curve in Figure 2, middle column, bottom row), confirming its diffusion to the interfacial area. Note that this absorption band is sharply cut off by the minimum wavelength of the detector sensitivity range at ca. 300 nm. Once more, as also observed at the intermediate OCP, an absorption band with a peak maximum at 373 nm was observed after 20 min and attributed to the absorption of the TB^–^ anion likely present as a BA^+^TB^–^ ion pair.

The influence of the polarization of the aqueous–TFT interface on the SERS spectra obtained at interfacial AuNP films in the presence of the aqueous and organic background electrolyte ions only was also investigated [Figure 2 (right column)]. Furthermore, each panel reports the reference spectra of the organic electrolyte salts used to set each OCP condition in the powder form. For each OCP condition, the Raman peaks of the organic ions (BA^+^ or TB^–^) in the SERS spectra obtained from the interfacial AuNP films in situ at the polarized aqueous–TFT interface were intense. Conversely, in the absence of the interfacial AuNP film, no Raman peaks were detectable at the polarized aqueous–TFT interface except those of TFT (Figure S13, Supporting Information). These results confirmed the SERS effect at interfacial AuNP films formed at each OCP condition. Previously, Booth et al. observed a strong SERS signal from BA^+^ using the reversible interfacial adsorption of citrate-stabilized silver nanoparticles (AgNPs) at an aqueous-1,2-dichlorobenzene interface.^49^ The interfacial adsorption of the AgNPs, and thus the SERS effect, only occurred at negative  values (controlled by external electrochemical polarization of the liquid–liquid interface using a 4-electrode electrochemical cell), allowing the generation of an on/off SERS effect. By contrast, herein, the AuNPs were irreversibly assembled at the aqueous–TFT interface, with the resulting SERS effect being present at OCP values covering the full range of the PPW.

### Detecting Tolmetin Using the SERS Effect in the Presence of an Interfacial AuNP Film at the Aqueous–TFT Interface

**Figure 3:**
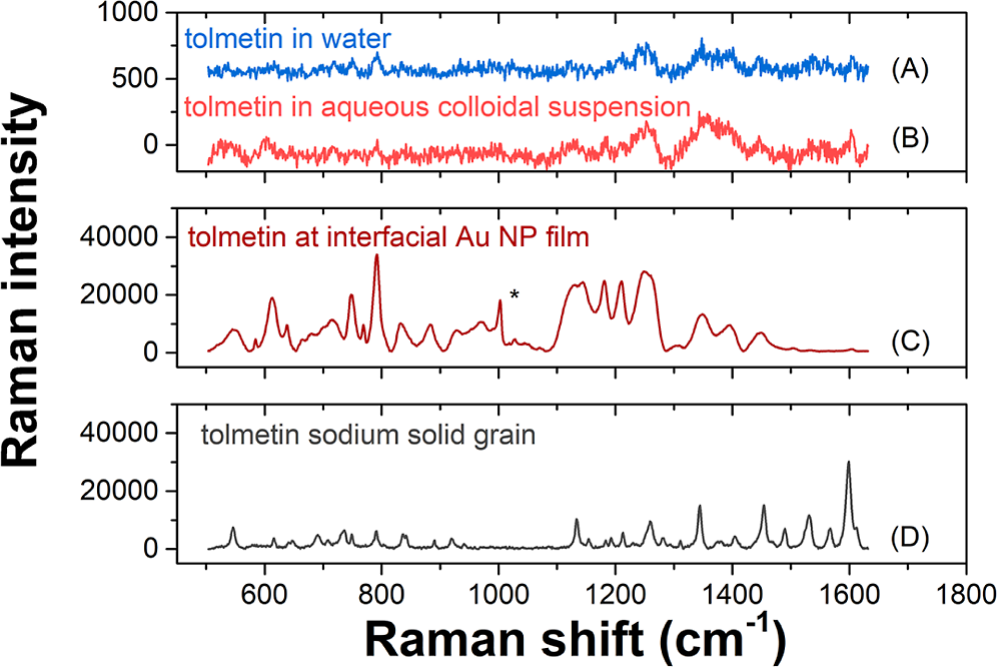
Raman spectra of (a) 1 mM tolmetin in water, (b) 1 mM tolmetin in an aqueous suspension of colloidal AuNPs, (c) 1 mM tolmetin at the interfacial AuNP film formed at the intermediate OCP set using the BATB electrolyte, and (d) a solid grain of tolmetin sodium as a reference spectrum. Raman spectra in (a) and (b) were acquired using a 532 nm laser at 100% power output (200 mW). The Raman spectra in (c) was acquired using a 785 nm laser at 1% power output (5 mW). Note that spectrum (d), acquired using a 532 nm laser at 1% (2 mW), is intense since it analyzes a pure powder with a very high local concentration of tolmetin molecules. The asterisk symbol in (c) indicates a Raman peak of the TFT solvent.

Raman spectra were obtained from single-phase experiments of tolmetin in water or in an aqueous colloidal suspension of AuNPs, a biphasic experiment where tolmetin was present at the interfacial AuNP film formed at the intermediate OCP set using BATB electrolyte, and from a solid grain of tolmetin sodium as a reference spectrum (Figure 3). Raman spectra from the single-phase experiments were acquired using a 532 nm laser at 100% power output. This laser wavelength corresponds with the λ_SPR_ of colloidal AuNPs (see Figure 1B) and was thus predicted as the optimal wavelength for Raman enhancement with a stable colloidal AuNP suspension. Raman spectra from the interfacial AuNP film were acquired using a 785 nm laser at 1% power output. In this case, the laser wavelength corresponds with the interfacial AuNP film plasmonic band that is strongly shifted to the near-infrared region compared with the λ_SPR_ of the colloidal AuNP suspension [Figure 2 (middle column)].

The Raman cross-section of tolmetin at 532 nm is low because it lacks electronic resonance enhancement as tolmetin does not absorb in the visible range. In this regard, the Raman spectrum of tolmetin in water demonstrates the limitations of conventional Raman spectroscopy for detecting tolmetin at concentrations of 1 mM or lower (Figure 3A), with a poor signal-to-noise ratio observed despite a 100% power output of the laser being applied to the solution. The Raman spectrum of tolmetin in the presence of a colloidal AuNP suspension displayed no SERS effect (Figure 3B). Tolmetin has a p*K*_a_ of 3.5 (see the chemical structure in Figure S14, Supporting Information)^50^ and was thus deprotonated and anionic under the aqueous phase conditions employed of ca. pH 5.5. As a result, chemical adsorption of tolmetin on the AuNPs was inhibited due to the electrostatic repulsive force between the negatively charged tolmetin and citrate-capped AuNPs. Consequently, no aggregation of the AuNPs occurred and no SERS signal could be generated. The lack of aggregation of the AuNPs was corroborated by the absence of a change of color of the solution upon the addition of tolmetin to the colloidal AuNP suspension.

The high intensity and signal-to-noise ratio of the spectrum shown in Figure 3C confirmed that a significant Raman enhancement, i.e., a SERS effect, occurs for tolmetin in the presence of the interfacial AuNP film formed at the intermediate OCP. The observation of a SERS effect indicates that tolmetin adsorbed on the interfacial AuNP films in spite of the electrostatic repulsive force between the negatively charged tolmetin and citrate-capped AuNPs, discussed vide supra. This suggests that the method in which the interfacial AuNP film is formed at the liquid–liquid interface, owing to the use of a high concentration of NaCl to initiate the aggregation process, facilitates the adsorption of tolmetin on the AuNP film, as illustrated in Scheme 2. Most of the Raman peaks detected for tolmetin in the presence of the interfacial AuNP film (Figure 3C) are similar to those obtained for the solid crystal grain of tolmetin sodium (Figure 3D). However, some significant spectral changes were observed. For example, the relative intensity of the peaks varied and the peak at 1600 cm^–1^, prominent in the solid spectrum in Figure 3D, is almost missing in the spectrum in the presence of the interfacial AuNP film in Figure 3C. Furthermore, an additional peak due to the aromatic ring breathing symmetric stretching of the TFT solvent is also visible at 1004 cm^–1^ in Figure 3C.

### Potential-Modulated SERS Spectra of Tolmetin in the Presence of Interfacial AuNP Films

**Figure 4:**
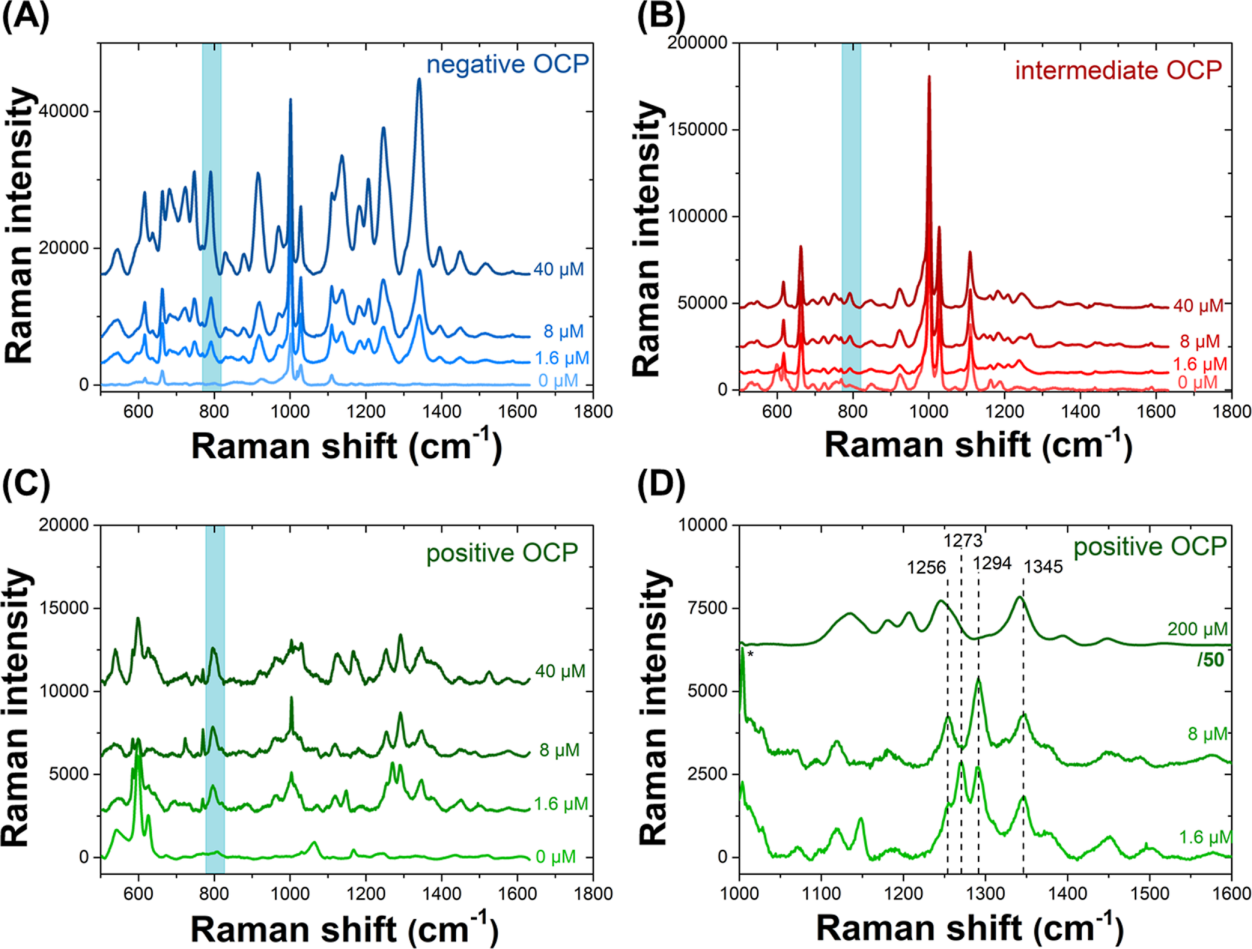
SERS spectra of tolmetin at interfacial AuNP films formed at (a) negative, (b) intermediate, and (c, d) positive OCPs. For panels (a)–(c), the tolmetin concentrations were 0, 1.6, 8, and 40 μM, respectively. The blue box highlights the band at 792 cm^–1^ common at all OCP conditions. In panel (d), a detailed view of the SERS spectra of 1.6, 8, and 200 μM tolmetin shows that the Raman spectra changed significantly between 1000 and 1600 cm^–1^. All Raman spectra were acquired using a 785 nm laser at 1% power output (5 mW).

SERS spectra of increasing concentrations of tolmetin, at an initial concentration of up to 1 mM in the aqueous phase, were obtained in the presence of interfacial AuNP films formed at negative, intermediate, and positive OCPs set by BACl, BATB, and LiTB electrolytes, respectively. Due to the SERS effect, the detection of initial tolmetin concentrations in the aqueous phase as low as 1.6 μM was possible at each OCP, as shown in Figure 4. Furthermore, the Raman spectra of tolmetin was potential-modulated, showing different characteristics depending on the OCP, as shown in Figure 4A–C for low (micromolar) tolmetin concentrations and Figure S15, Supporting Information, for a higher 1 mM tolmetin concentration.

At the negative OCP set using the BACl electrolyte, the Raman spectra increased in intensity as the tolmetin concentration increased but otherwise remained unchanged, with the relative intensities and positions of the Raman peaks remaining constant throughout the concentration range. By comparison, at the positive OCP set using the LiTB electrolyte, the features of the Raman spectra between 1000 and 1600 cm^–1^ changed significantly from 1.6 to 200 μM, as shown in Figure 4D. At 1.6 μM, four features were observed in this range: a shoulder at 1256 cm^–1^ and three peaks at 1273, 1294, and 1345 cm^–1^. The peak at 1273 cm^–1^ disappeared at 8 μM, and the peak at 1294 cm^–1^ disappeared at 200 μM tolmetin, leaving only two peaks around 1250 and 1345 cm^–1^. The vibrational modes of tolmetin calculated by density functional theory (DFT) corresponding to these experimental frequencies, and several others, are summarized in Table S1, Supporting Information, and shown in an animated GIF in Movie S1, Supporting Information. A comparison of the calculated frequencies and those experimentally obtained for tolmetin is shown in Figure S16, Supporting Information. The fact that the mode at 1273 cm^–1^, corresponding to a pyrrole ring stretching coupled to a stretching mode of the phenyl ring, was enhanced at the lowest tolmetin concentration (1.6 μM) but becomes negligible ≥8 μM is probably related to a change of the adsorption geometry as a function of the rate of coverage of the AuNP surface by the adsorbing tolmetin molecule. As the aqueous tolmetin concentration increased in the aqueous phase, so too did the number of adsorbed molecules on the AuNPs, and this may have changed the way the tolmetin molecules interact with the AuNP surface due to the steric hindrance or the development of intermolecular forces. At an intermediate OCP, the Raman spectra are mainly dominated by the SERS of background electrolyte molecules.

### Probing the Limits of the Use of Potential-Modulated SERS at an Interfacial AuNP Film as a Quantitative Analytical Tool for Tolmetin

The relationship between the SERS peak intensity and the concentration of the molecule analyzed is often nonlinear.^51^ While a range where SERS intensity is proportional to the decimal logarithm of concentration may be found, this relationship is rarely valid over a truly extended concentration range.^52^ Experimentally, curves may be observed that are in effect several successive linear regimes. Bell-shaped curves, showing a decline in SERS intensity after a certain concentration of the analyte, may also be observed. Modeling of these phenomena implies an attenuation of surface plasmons by molecules adsorbed on metal nanoparticles, known as chemical interface damping (CID), and is associated with a change in the adsorption regime when moving from a monolayer to a multilayer of molecules.^53^

**Figure 5:**
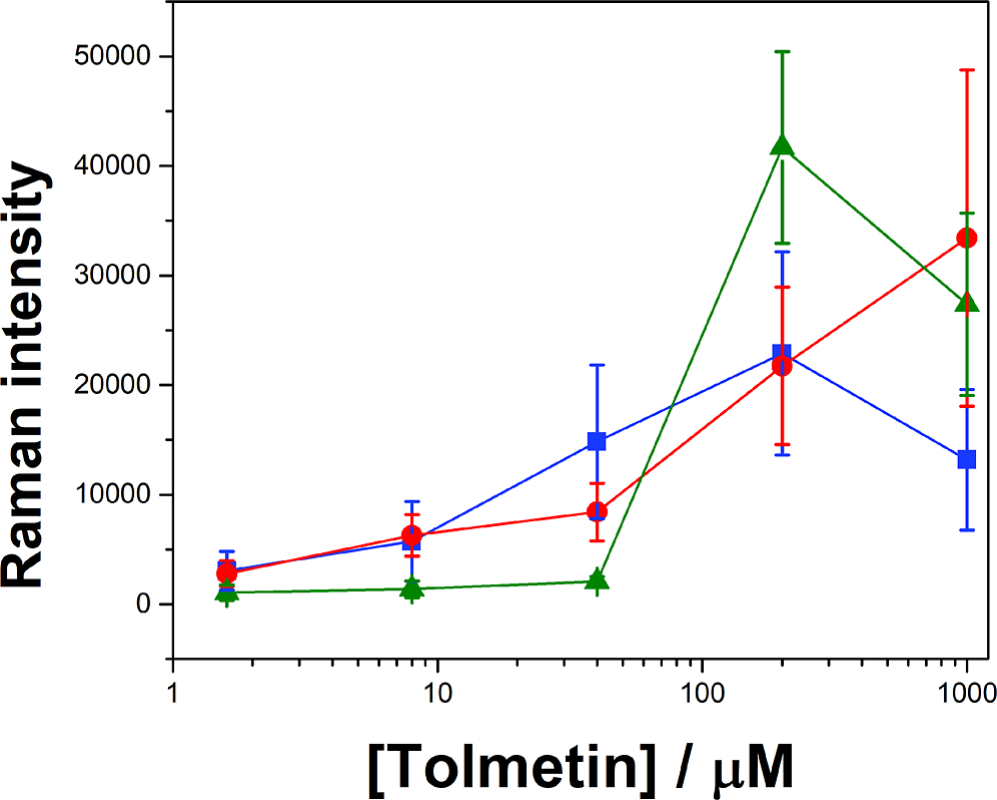
Raman intensity of a characteristic peak of tolmetin at 792 cm^–1^ as a function of the initial tolmetin concentration in the bulk aqueous phase at a negative (■), intermediate (●), and positive (▲) OCP.

To probe the limits of the use of potential-modulated SERS at an interfacial AuNP film as a quantitative tool, we measured the Raman intensity of a characteristic peak of tolmetin at 792 cm^–1^ (highlighted in blue in Figure 4A–C) that did not disappear or undergo a chemical shift when the interfacial polarization was changed. DFT calculations show that this peak is principally due to a phenyl ring symmetric deformation, see Table S1, Supporting Information. The Raman intensities of this peak versus the initial aqueous tolmetin concentration at negative, intermediate, and positive OCPs are shown in Figure 5. The Raman intensity at 792 cm^–1^ increased in magnitude at each OCP condition as the initial tolmetin concentration in the aqueous phase increased up to 200 μM. However, none of these increases in magnitude were linear in nature. For lower concentrations of tolmetin ≤40 μM, the Raman intensity was the highest at the negative OCP, followed by the intermediate, and positive OCPs. Subsequently, the trend reversed at 200 μM, and the Raman intensity was the highest at the positive OCP. Furthermore, at tolmetin concentrations >200 μM, the Raman intensity decreased at the positive and negative OCPs. An intriguing observation is that the error bars tend to enlarge as the concentration increases. This phenomenon indicates that a higher SERS intensity correlates with the formation of a greater number of active hot spots. Consequently, there is an amplification in the variability of intensity. This variability is attributed to the electromagnetic enhancement being significantly affected by various local factors. These include the nanoscale roughness of surfaces, the proximity of hot spots within the fractal films, and the orientation of the local surface relative to the incident laser’s polarization direction. Such effects are diminished when the SERS intensity is at a lower level. These trends in Raman intensity as a function of the initial tolmetin concentration in the aqueous phase with OCP are due to an interplay of the following:

- the adsorption of tolmetin molecules on the AuNP clusters formed in the bulk aqueous phase during interfacial AuNP film assembly, independent of the applied OCP,
- the changes in the morphology of the interfacial AuNP films, and associated changes in the available surface area for tolmetin molecules to adsorb and nature of the SPR band, as a function of the applied OCP,
- the variation of the concentration of anionic tolmetin in the interfacial region, in comparison to the initial bulk aqueous concentration, as a function of the applied OCP, and
- CID of the plasmons of the interfacial AuNP film above a threshold initial bulk aqueous concentration of tolmetin, independent of the applied OCP.

Each of these points is addressed separately in the following paragraphs and, their interplay at each OCP, ultimately leading to the Raman intensities observed in Figure 5, is then outlined.

As noted vide supra, the method in which the interfacial AuNP films were formed at each OCP facilitates the adsorption of tolmetin on the AuNP film (while tolmetin does not adsorb on colloidal AuNPs in suspension). The initial step in the formation of the interfacial AuNP films was the addition of a high concentration of NaCl to the bulk aqueous phase, during which the large concentration of Na^+^ cations in solution neutralized some of the negative charge on the citrate-stabilized AuNPs. This induced the formation of small clusters of AuNPs in the bulk aqueous phase but also facilitated the interaction and adsorption of negatively charged tolmetin molecules with the AuNP clusters, see Scheme 2. This process was independent of the polarization of the aqueous–TFT interface as it only occurred in the bulk aqueous phase and, thus, contributed to the Raman intensity at 792 cm^–1^ increasing in magnitude as the initial tolmetin concentration in the aqueous phase increased up to 200 μM at each OCP condition. The SERS effect is possible because tolmetin molecules are trapped within the AuNP clusters that form the film at the interface. If the AuNP film is formed in the absence of tolmetin and tolmetin is added to the aqueous phase after the film formation, no SERS effect was observed (Figure S17, Supporting Information).

As discussed in detail vide supra, due to the changes of the morphology of the interfacial AuNP film as a function of the OCP -Figure 2 (left column)], an inference can be made that the gold surface area available for tolmetin to adsorb decreases going from a negative to intermediate to positive OCP, respectively. Furthermore, as shown in Figure 2 (center column), the absorbance of the SPR band at 785 nm is moderate at the intermediate and negative OCPs but strong at the positive OCP.

Electrochemical experiments using a 4-electrode electrochemical cell provide additional information that the concentration of anionic tolmetin in the interfacial region increases at the negative OCP set by BACl. Cyclic voltammograms obtained in the presence of increasing concentrations of tolmetin in the aqueous phase clearly show a shortening of the PPW at the negative extreme as tolmetin molecules are driven to the interface and transfer from the aqueous phase to organic phase at negative  values (see Scheme 2 and Figure S18, Supporting Information). On the contrary, at the intermediate OCP set by BATB, the tolmetin concentration in the interfacial region should be similar to that in the bulk aqueous phase, while at the positive OCP set by LiTB, a depletion of tolmetin concentration in the interfacial region is expected and ion transfer of tolmetin from the aqueous phase to organic phase is entirely inhibited.

When more than a monolayer of tolmetin molecules cover the exposed gold surface in an interfacial AuNP film, it leads to CID, weakening the plasmonic waves and reducing the electromagnetic enhancement of the Raman signal. To probe this effect, the “real” concentration of tolmetin in the interfacial AuNP film was determined and used to estimate whether a submonolayer, monolayer, or multilayer(s) of tolmetin molecules were adsorbed on the exposed gold surface. To achieve this, knowing the initial tolmetin concentration in the bulk aqueous phase, UV–vis absorption spectroscopy was used to determine the concentrations of tolmetin in the aqueous and organic phases after the biphasic system was equilibrated for a period of 5 h. From these data, the “real” concentration of tolmetin in the interfacial AuNP film was determined and converted into an estimate of the number of tolmetin molecules adsorbed per nm^2^ of gold surface. The latter calculation took into the account the number of AuNPs per mL in the aqueous phase prior to interfacial AuNP film formation [determined to be 1.48 (±0.09) × 10^11^ AuNPs mL^–1^, see Figure S3, Supporting Information] and made assumptions regarding the geometry of the AuNPs in the aggregates or clusters that form the interfacial AuNP film.

An estimation of the number of tolmetin molecules adsorbed per nm^2^ of gold surface was only possible for the interfacial AuNP films formed at the negative OCP (Figures S19–S21, Supporting Information) as at the intermediate and positive OCPs, the absorption band of TB^–^ in the organic phase [peak maximum at 373 nm, see Figure 2 (middle column)] overlapped with that of tolmetin (peak maximum at 316 nm, see Figure S20, Supporting Information). At the negative OCP, an assumption was made that aggregates were formed by AuNPs in relatively close contact with each other, but each AuNP still retained its spherical geometry. While this assumption certainly led to an underestimation of the number of tolmetin molecules adsorbed per nm^2^ of gold surface, as aggregates typically diminish the gold surface area available for the adsorption process, it still allowed an order of magnitude to be estimated. As summarized in Table S2, Supporting Information, a submonolayer of tolmetin (0.5 molecules per nm^2^) was only estimated for the lowest initial tolmetin concentration in the bulk aqueous phase investigated of 8 μM, while at the higher tolmetin concentrations of 40, 200, and 1000 μM, multilayers were formed with 3, 43, and 264 molecules per nm^2^, respectively. For these higher tolmetin concentrations, the adsorption mechanism differed, and at a certain threshold concentration, the distance between the SERS hot spots and tolmetin molecules increased to a tipping point, beyond which the SERS efficiency decays exponentially with distance.^54,55^ This contribution, in addition to the possible CID of the plasmons, explains why the Raman intensity decreased beyond a certain initial tolmetin concentration in the bulk aqueous phase.

While the concentration of tolmetin could not be determined in the organic phase after the biphasic system equilibrated at the intermediate and positive OCPs, as noted vide supra, the concentration in the aqueous phase was determined (Figure S22, Supporting Information). For comparison, 15, 95, and 75% of the tolmetin molecules initially present in the bulk aqueous phase remained after equilibration at the negative, intermediate, and positive OCPs, respectively (as determined by UV–vis absorption spectroscopy for an initial tolmetin concentration of 40 μM, see Figure S22 and Tables S2 and S3, Supporting Information). Anionic tolmetin undergoes ion transfer to the organic phase at the negative OCP, explaining the low concentration remaining in the aqueous phase. Meanwhile, as no thermodynamic driving force was present to transfer tolmetin to the organic phase at intermediate and positive OCPs, any reduction of tolmetin concentration in the aqueous phase compared to that initially present was primarily due to its absorption onto the interfacial AuNP film. Thus, these results indicate that more tolmetin was adsorbed on the interfacial AuNP film formed at the positive OCP (25%) than that at the intermediate OCP (5%), possibly due to the differences in morphology between each film, the nature of the electrolyte salts presents in the film, and the interfacial polarization.

To summarize the trends in Figure 5, the interfacial AuNP film formed at the negative OCP shows a better performance to detect lower (1.6–40 μM) initial tolmetin concentrations in the aqueous phase. While the absorbance of the SPR band at 785 nm is moderate, the concentration of tolmetin in the interfacial region and the gold surface area available for tolmetin to adsorb are both enhanced at the negative OCP. On the other hand, the interfacial AuNP film formed at the positive OCP shows a better performance to detect higher (200 μM) initial tolmetin concentrations in the aqueous phase. While the absorbance of the SPR band at 785 nm is strong, the concentration of tolmetin in the interfacial region and the gold surface area available for tolmetin to adsorb are both diminished at the positive OCP. In other words, the enhanced accumulation of tolmetin molecules on the gold surface at the negative OCP is advantageous at low initial tolmetin concentrations, while the strongly absorbing plasmon band overcomes the poor accumulation effect at the positive OCP at high initial tolmetin concentrations. For tolmetin concentrations >200 μM, the SERS signal decreased sharply at the negative and positive OCPs due to the CID of the plasmons of the interfacial AuNP film. No CID effect was observed at the intermediate OCP, possibly due to a lower number of molecules per nm^2^_,_ as inferred by the low loss (just 15%) of tolmetin molecules initially present in the bulk aqueous phase after equilibration of the biphasic system (Figure S22, Supporting Information).

## Conclusions

In this article, we formed fractal gold films at a polarizable liquid–liquid interface from aqueous AuNP suspensions. The morphology of these films is controlled by modifying the polarization at the interface between the two liquids. These gold films feature a surface roughness compatible with the plasmonic exaltation effect in SERS using a visible laser wavelength at 785 nm.

These gold films are shown to be suitable for SERS spectroscopic detection of molecules of interest that are electronically nonresonant with the laser wavelength. In particular, we have demonstrated the SERS effect with tolmetin, a negatively charged molecule in solution under our working conditions. This work highlights the importance of using a “soft” liquid–liquid interface as it enables the detection of molecules in an aqueous environment, paves the way to study the influence of parameters such as pH or ionic strength on the SERS signal, and prevents degradation of the analyte molecule by any laser heating effect by avoiding the surface local heating of SERS substrates used in dry conditions.

The inherent location of the fractal gold film at the liquid–liquid interface facilitates its analysis by Raman spectroscopy using an immersion objective. The simplicity of the experiment, which consists in bringing two immiscible solutions into contact and waiting for the film to form as sodium chloride is added to the aqueous phase, is a major advantage of this approach and enhances the reproducibility of the obtained signals. The stability of fractal gold films at the liquid–liquid interface allows the Raman mapping of the interface, yielding numerous spectra and strongly strengthening the statistical significance of the data. This is a crucial step toward exploiting this approach for analytical purposes.

Future work will involve extending this approach to a broad spectrum of organic molecules, with a preference for those with environmental significance. Furthermore, attempting to alter the polarization of the interface by applying a well-controlled potential will enable us to enhance discrimination and detection capabilities. The application of this method could also be directed toward detecting biomolecules at polarized interfaces, such as viral capsids, and exploring their contents, including genetic information (DNA or RNA) as well as proteins.

## Supporting Information Available

Supplementary experimental methods; synthesis, characterization, and aggregation of the AuNPs; polarization of the aqueous–TFT interface and associated morphological changes of the interfacial AuNP films; Raman spectroscopy; electrochemical behavior of tolmetin at the polarizable aqueous–TFT interface; and determination of tolmetin biphasic distribution by UV–vis spectroscopy (PDF)
Vibrational modes of tolmetin (MP4)
