# Supplementary material for: Potential-Modulated Surface-Enhanced Raman Spectroscopy of Tolmetin at Gold Nanoparticle Film Functionalized Polarizable Liquid–Liquid Interfaces
Source: J Phys Chem C Nanomater Interfaces. 2024 May 4;128(19):7936–47. doi: 10.1021/acs.jpcc.4c00937 (PMC11103698; doi:10.1021/acs.jpcc.4c00937)
Supplement: Supplementary file 1 — jp4c00937_si_001.pdf [file jp4c00937_si_001.pdf]

Supporting Information

Potential Modulated Surface-enhanced Raman Spectroscopy of Tolmetin at Gold Nanoparticle Film Functionalised Polarisable Liquid-Liquid Interfaces

Madjid Tarabet,[a] Nataly Rey Muñoz,[b] Micheál D. Scanlon,[b]* Grégoire Herzog,[a]* Manuel Dossot[a]*

[a] Université de Lorraine, CNRS, LCPME, F-54000 Nancy, France.

[b] The Bernal Institute and Department of Chemical Sciences, School of Natural Sciences, University of Limerick (UL), Limerick V94 T9PX, Ireland.

* Corresponding authors: micheal.scanlon@ul.ie, gregoire.herzog@cnrs.fr, manuel.dossot@univ-lorraine.fr.

Table of Contents

Section	Title	Page
**S1.**	**Supplementary Experimental Methods**	S3
S1.1	*In situ* and *ex situ* materials characterisation techniques	S3
S1.2	Electrochemistry at a polarisable liquid-liquid interface using a 4-electrode electrochemical cell	S4
**S2.**	**Synthesis, characterisation, and aggregation of the AuNPs**	S6
S2.1	AuNP synthesis	S6
S2.2	AuNP characterisation	S6
**S3.**	**Polarisation of the aqueous-TFT interface and associated morphological changes of the interfacial AuNP films**	S12
S3.1	Open circuit potential (OCP) measurements	S12
S3.2	SEM images of the morphology of the interfacial AuNP films in the absence and presence of 1 mM tolmetin as a function of the polarisation of the aqueous-TFT interface	S13
S3.3	Fractal dimensions of the morphology of the interfacial AuNP films in the presence and absence of 1 mM tolmetin as a function of the polarisation of the aqueous-TFT interface	S15
**S4.**	**Raman spectroscopy**	S18
**S5.**	**Electrochemical behaviour of tolmetin at the polarisable aqueous-TFT interface**	S25
**S6.**	**Determination of tolmetin biphasic distribution by UV-vis spectroscopy**	S26
S6.1	Single phase experiments: calibration curves of tolmetin in the aqueous and organic phases, respectively	S26
S6.2	Biphasic experiments: UV-vis absorption spectra of tolmetin in the aqueous and organic phases, respectively, at equilibrium	S28
**S7.**	**Supplementary references**	S31

S1. Supplementary Experimental Methods

S1.1 *In situ* and *ex situ* materials characterisation techniques

UV-vis absorption spectroscopy. To characterise the gold nanoparticles (AuNPs) synthesised and for the determination of tolmetin concentration in both the organic and aqueous phases, a Carry 6000i dual-beam UV-vis-NIR spectrophotometer with 1 cm pathlength quartz cuvettes was used to obtain UV-vis absorption spectra. A baseline with solvent-filled cuvettes in the reference and sample holders was obtained before measurements. The solvent-filled reference cuvette was used as a standard. The settings were: 1 nm of spectral resolution, 400 $nm \cdot min^{-1}$ for scanning speed, and 0.5 nm data interval.

***In situ* UV-vis absorption spectroscopy at the polarisable liquid-liquid interface.** Interfacial AuNP film assembly was investigated by *in situ* UV-vis absorption spectroscopy in either transmission or total internal reflection (TIR) detection modes. The spectrometer used was a USB 2000 Fiber Optic Spectrometer (Ocean Optics). The light source was a HPX-2000 Xenon light (Ocean Optics), which was guided through an optical fibre with a diameter of 600 μm (Ocean Optics, USA). In transmission detection mode, the light beam was collimated using optical lenses (Thorlabs, focal length: 2 cm) before and after the transmission of the beam through both the organic and aqueous phases. In TIR detection mode, the light beam was collimated to pass through the interface to probe the plasmonic properties of the assembled AuNP film.

Dynamic light scattering (DLS) and zeta (ξ)-potential measurements. Hydrodynamic size and ξ-potential characterisations of the synthesised AuNPs were carried out using a capillary cell (Malvern Instruments, DTS1061) at room temperature, employing the Zetasizer Nano ZS (Malvern Instruments) with a red He-Ne laser (λ = 633 nm), and operated with Dispersion Technology Software (Malvern Instruments, DTS). The size distribution of the AuNPs was assessed by DLS, where the diffusion coefficient was converted into size using the Stokes-Einstein equation. The ξ-potential of the AuNPs was estimated by measuring the intensity of repulsion and electrostatic attraction between particles, occurring between the two electrodes of the cell.

Transmission electron microscopy (TEM). TEM images of the synthesised AuNPs were obtained by depositing a small volume of a diluted colloidal AuNP suspension in ethanol onto a copper grid (Gilder Grids LTD). The micrographs were acquired using the Philips CM20

transmission electron microscope with an acceleration voltage of 200 kV. The AuNP size distributions were calculated using Image J (https://imagej.nih.gov/ij/download.html).

Scanning electron microscopy (SEM). AuNP films formed at the polarised aqueous-α,α,α-trifluorotoluene (TFT) interface were subsequently transferred onto TEM copper metal substrates, which had been positioned on a stainless-steel grid at the bottom of the organic phase. Following film formation, the organic phase was delicately removed, allowing the film to adhere to the TEM grid. Once deposited and dried, SEM analysis was conducted. The AuNP film morphology was examined using a JEOL JSM-IT500 HR scanning electron microscope equipped with a field emission gun (FEG) and an Energy-Dispersive X-ray Spectrometer (EDS). The analyses were performed under a high vacuum level, with a 60 μm diaphragm to limit interference. The acceleration voltage for the analyses was set at 10 kV. The fractal dimensions of the AuNP films were determined using ImageJ software (details *vide infra*).

Raman spectroscopy. A Renishaw Qontor confocal Raman microscope was used with a 785 nm solid laser (500 mW) or a 532 nm laser (200 mW), a 1200 grooves·mm^{-1} grating (785 nm) or a 2400 grooves·mm^{-1} grating (532 nm), and a Peltier cooled CCD camera to acquire Raman spectra. For solid samples, a × 50 long working distance objective (0.50 numerical aperture) was used and the laser power reduced below 1% to prevent laser damage. We averaged 5 spectra of 4 s each, with 1% power of the lasers.

For *in situ* Raman spectroscopy at a polarised aqueous-TFT interface, a × 60 water immersion objective (0.90 numerical aperture) was used. 1% laser power was used to collect the surface enhanced Raman spectroscopy (SERS) signal, with the same acquisition conditions as for solid samples. Raman mapping analysis on the AuNP films formed at a polarisable aqueous-TFT interface were performed, which had high quality and stability. This allowed us to collect 88 spectra from different locations, enhancing the statistical significance of the results. The spectra shown in this manuscript are the mean spectra of 88 individual spectra.

S1.2 Electrochemistry at a polarisable liquid-liquid interface using a 4-electrode electrochemical cell

All electrochemical experiments were conducted using a four-electrode electrochemical cell setup. The surface area of the interface was 1.13 cm^2. Two platinum counter-electrodes were used, immersed in the aqueous phase and organic phases, respectively. Additionally, two silver wires coated with a layer of silver chloride served as reference electrodes (Ag/AgCl) immersed in the aqueous phase and organic reference phase of 100 mM NaCl and 2.5 mM

bis(triphenylphosphoranylidene)ammonium chloride (BACl). The composition of the organic phase remained constant throughout all electrochemical experiments, with 2.5 mM of bis(triphenylphosphoranylidene)ammonium tetrakis(pentafluorophenyl)borate (BATB) dissolved in TFT. The aqueous phase was composed of 100 mM NaCl, with different concentrations of tolmetin added.

Electrochemical measurements, including cyclic voltammetry (CV) and Alternating Current (AC) voltammetry, were carried out using an Autolab PGSTAT 302N potentiostat equipped with a FRA2 frequency modulator. The measurements were controlled using the NOVA 2.1.4 software developed by Metrohm Autolab. The standard transfer potential of tetramethylammonium (TMA^+, $\Delta_o^w \phi^0_{TMA^+} = 0.311$ V)[1] was used as a reference to calibrate the potential to the Galvani scale.

S2. Synthesis, characterisation, and aggregation of the AuNPs

S2.1 AuNP synthesis

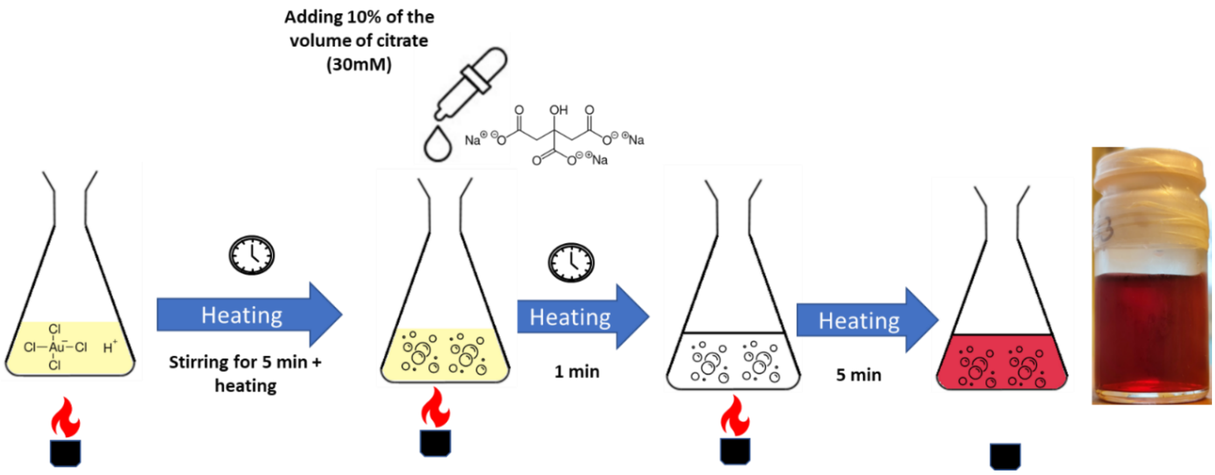

Fig. S1. Synthesis of AuNPs capped with citrate molecules using the revised Turkevich method[2] with modified citrate concentrations. The image on the right is of a colloidal suspension of the AuNPs.

For the synthesis of AuNPs, we took inspiration from the revised Turkevich method,[2]an approach based on the reduction of Au^{3+} ions in solution by trisodium citrate, acting as both a reducing agent and a stabiliser. We adjusted the precursor and reducing agent concentrations, aiming for a final molar ratio of 3:1 between chloroauric acid and citrate, respectively. The goal was to achieve a suspension adequately stable in solution and yet easy to destabilise upon an increase of the ionic strength.

S2.2 AuNP characterisation

Once synthesised, each batch of AuNPs was initially characterised by UV-vis absorption spectroscopy. The UV-vis absorption spectra of 3 batches of AuNPs are shown in Fig. S2.

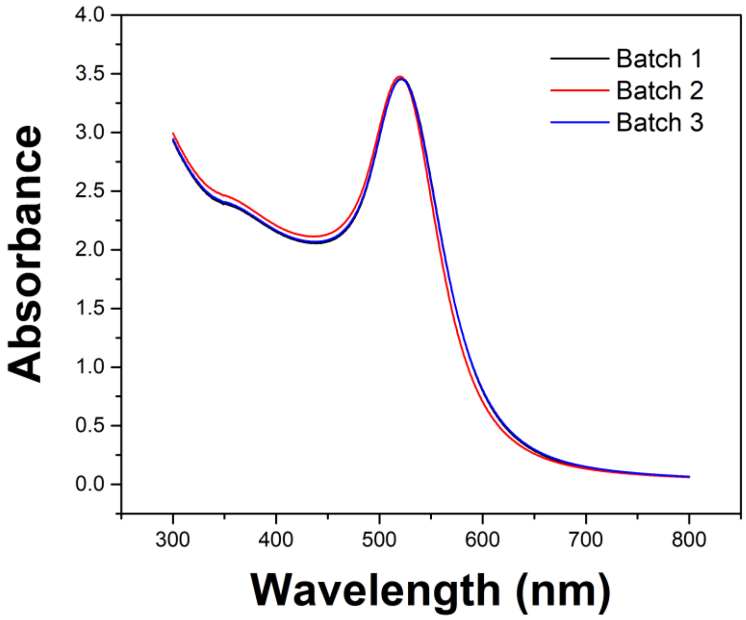

Fig. S2. UV-vis absorption spectra of 3 batches of citrate-capped AuNPs synthesised using the method described in Fig. S1.

The spectra in Fig. S2 are identical and suggest high reproducibility in AuNP size and concentration, *i.e.*, the number of AuNPs per mL of suspension (discussed *vide infra*), between batches. As described by Haiss *et al.*,[3] using Equation S1, the average diameter d in nanometres of the AuNPs suspended in water may be calculated by analysing the absorbance data at 450 nm (A_{450}) and at the maximum of the surface plasmon resonance band (A_{SPR}),

$$d = exp\left(B_1 \frac{A_{SPR}}{A_{450}} - B_2\right) \qquad \text{Equation (S1)}$$

Haiss *et al.* reported the experimentally determined fit parameters as $B_1 = 3.00$ and $B_2 = 2.20$, respectively.[3] Since A_{SPR} for the different batches of AuNPs was greater than 3, we considered the non-linear response of the spectrophotometer at such high absorbance values. The UV-vis absorbance spectra obtained for one batch of AuNPs, and the same batch diluted twice and five times, is shown in Fig. S3A. Plotting A_{SPR} *versus* the normalised concentration shows the deviation from linearity (Fig. S3B). Thus, the absorbance values for the diluted solutions were used to calculate the corresponding average diameter d using the uncertainty of the method reported.[3] The final average diameter given by Equation S1 is 22.0 ± 2.5 nm (this is the average of three batches).

The number of AuNPs mL^{-1} of suspension, N, may be calculated using Equation S2,[3]

$$N = \frac{A_{450} \times 10^{14}}{d^2\left[-0.295 + 1.36\, exp\left(-\left(\frac{d-96.8}{78.2}\right)^2\right)\right]}$$ Equation (S2)

Taking into account that d = 22 nm, as calculated above, the value of N was 1.48 (± 0.09) × 10^{11} AuNPs mL^{-1}.

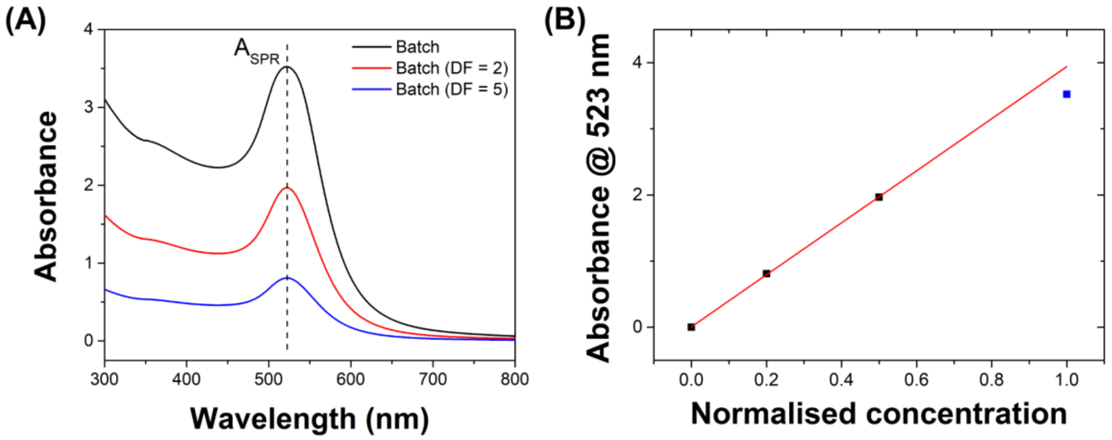

Fig. S3. (A) UV-vis absorbance spectra of citrate-capped AuNPs before and after dilution in water (DF means "dilution factor"). **(B)** Plot of A_{SPR} *versus* the normalised concentration. The absorbance value of the undiluted sample (blue data point) shows deviation from linearity.

A typical TEM micrograph at high magnification of the synthesised AuNPs is shown in Fig. S4A. The AuNP size distributions were calculated using Image J, with a count of the number of AuNPs *versus* their diameter performed for 3 TEM images from a single batch (Fig. S4B). A Gaussian distribution can be fitted to the data and gives the average diameter and the standard deviation of the population. The value of d from TEM analysis is 22 ± 5 nm.

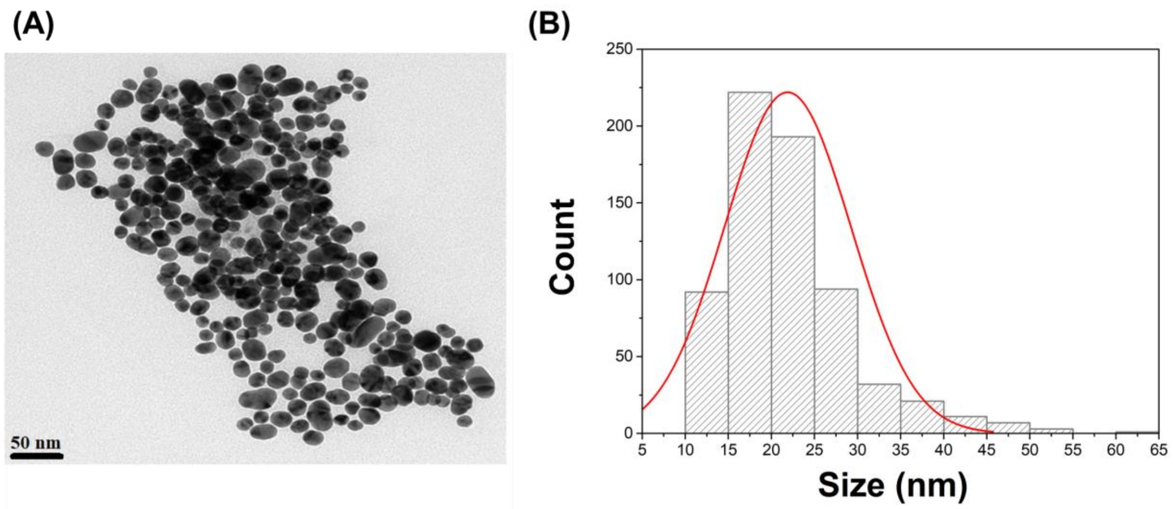

Fig. S4. **(A)** TEM micrograph of AuNPs dispersed in a mixture of water and ethanol (1:10). **(B)** Distribution of the diameters of AuNPs determined by TEM (*N* = 676 NPs using 3 TEM images from a single batch).

DLS was used to obtain the average hydrodynamic diameter for three batches of synthesised AuNPs (Fig. 5A). The average hydrodynamic diameter for the three batches is 27 ± 4 nm, in good agreement with the value obtain from UV-vis absorbance spectra (analysed using Equation S1) and the TEM analysis. The average ξ-potential of the AuNPs was determined as –44 ± 4 mV (Fig. S5B), confirming the capping of the surface of the AuNPs with negatively charged citrate molecules.

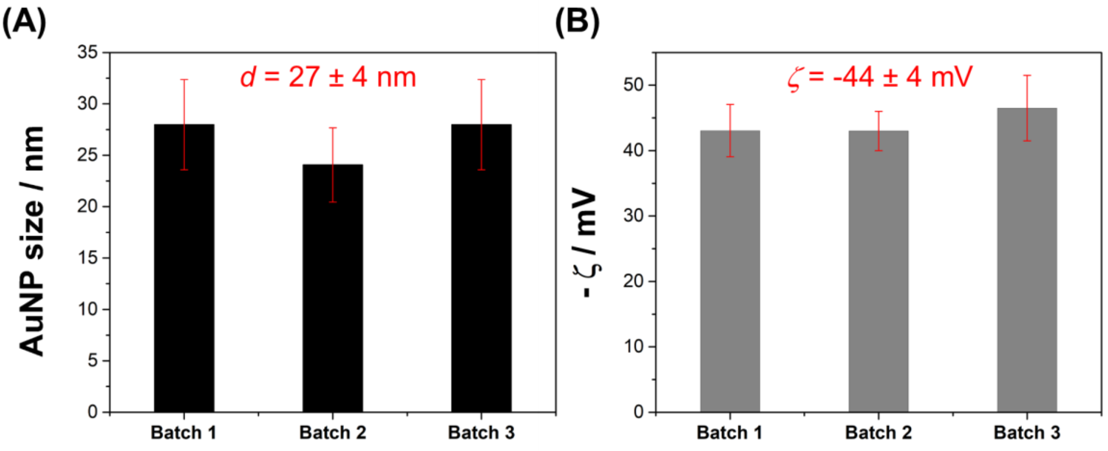

Fig. S5. (A) Average diameter, *d*, from DLS intensity-weighted size measurements and **(B)** zeta (ξ)-potential measurements of 3 different batches of synthesised AuNPs capped with negatively charged citrate molecules.

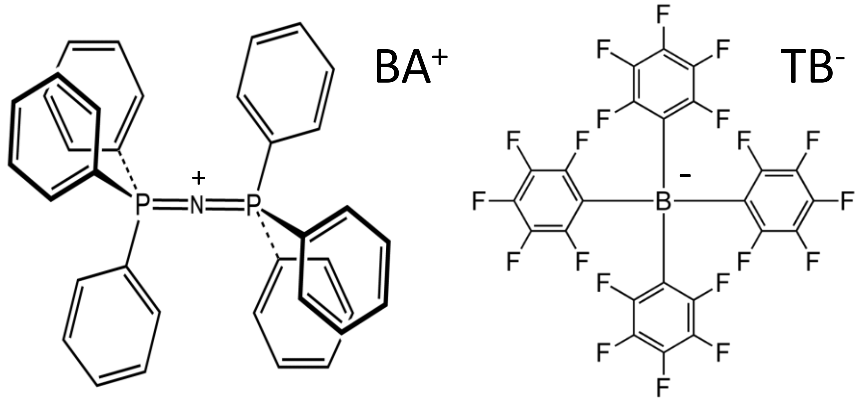

Fig. S6. Chemical structure of the organic background electrolyte salt bis(triphenylphosphoranylidene)ammonium tetrakis(pentafluorophenyl)borate (BATB).

DLS was used to characterise the aggregates formed by the addition of NaCl to the aqueous phase. Fig. S7 shows that the average size of AuNP aggregates increased as the NaCl concentration increased from to 5 to 100 mM, with a clear jump in the size of the AuNP aggregates formed at NaCl concentrations ≥ 50 mM. Note that the exact size of the aggregates cannot be determined by DLS since they sediment rapidly and, thus, the results of Fig. S7 are mainly qualitative.

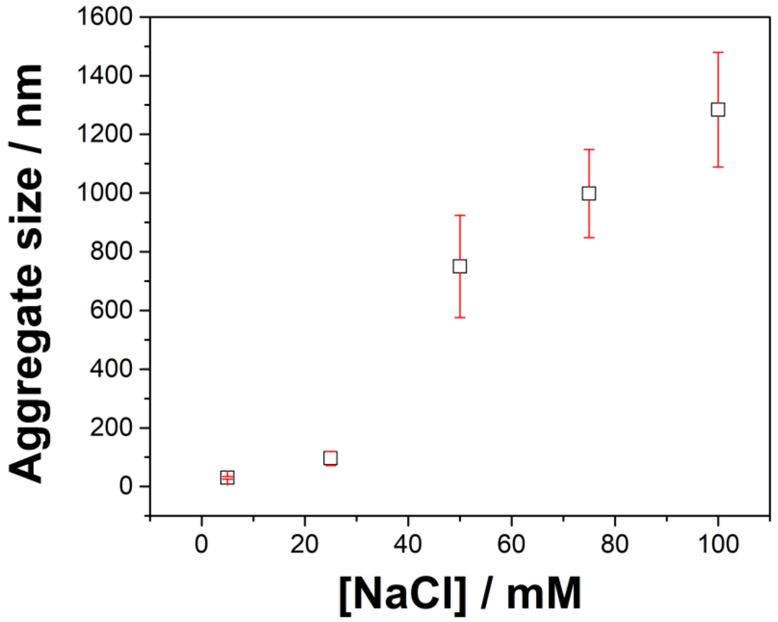

Fig. S7. Effect of NaCl concentration on the size of AuNP aggregates formed. Each point is the average size (obtained by DLS) of 3 different batches. The error bars represent their standard deviation.

S3. Polarisation of the aqueous-TFT interface and associated morphological changes of the interfacial AuNP films

S3.1 Open circuit potential (OCP) measurements

The OCP for the three different cells were measured in a four-electrochemical set-up using a Metrohm Autolab 302N potentiostats controlled by Nova 2.1.6 software. The three cells considered were the following:

Electrochemical cell 1 (for negative OCP): Ag | AgCl | 100 mM NaCl (aqueous phase) || 1 mM BACl (organic phase) | 1 mM BACl + 99 mM NaCl |AgCl | Ag

Electrochemical cell 2 (for intermediate OCP): Ag | AgCl | 100 mM NaCl (aqueous phase) || 2.5 mM BATB (organic phase) | 2.5 mM BACl + 97.5 mM NaCl |AgCl | Ag

Electrochemical cell 3 (for positive OCP): Ag | AgCl | 100 mM NaCl (aqueous phase) || 2.5 mM LiTB (organic phase) | 2.5 mM BACl + 97.5 mM NaCl |AgCl | Ag.

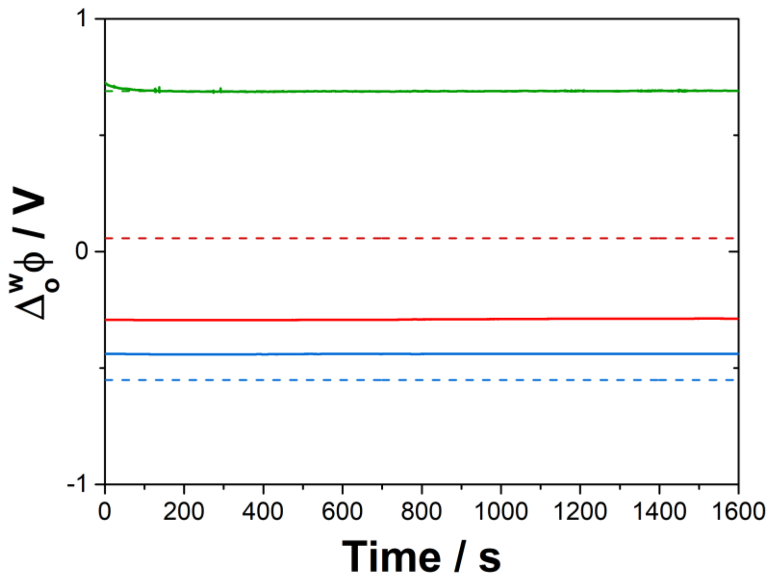

Fig. S8. Open circuit potential (OCP) measurements obtained for liquid-liquid interfaces prepared with an aqueous phase of 100 mM NaCl and an organic phase containing either 1 mM bis(triphenylphosphoranylidene)ammonium chloride (BACl, blue line), 2.5 mM BATB (red line), or 2.5 mM lithium tetrakis(pentafluorophenyl)borate diethyletherate (LiTB, green line) electrolyte salts in TFT solvent. Solid lines represent experimental measurements, while dashed lines represent calculated OCP values.

The expected OCP values (red and blue dashed lines) were calculated based on the previous works[4,5] and were used to calibrate the potential windows on the Galvani scale. For these calculations, the Gibbs energy of transfer used were the following: $\Delta_o^w G_{Na^+}^0$ = 75.9 kJ mol^{-1};[6] $\Delta_o^w G_{Cl^-}^0$ = 64.6 kJ mol^{-1};[6] $\Delta_o^w G_{BA^+}^0$ = -71.9 kJ mol^{-1}; $\Delta_o^w G_{TB^-}^0$ = -71.0 kJ mol^{-1}. Based on calculations, it is likely that a sub-micromolar contamination of BATB with BACl from the metathesis reaction leads to the "intermediate" OCP being so negative.

S3.2 SEM images of the morphology of the interfacial AuNP films in the absence and presence of 1 mM tolmetin as a function of the polarisation of the aqueous-TFT interface

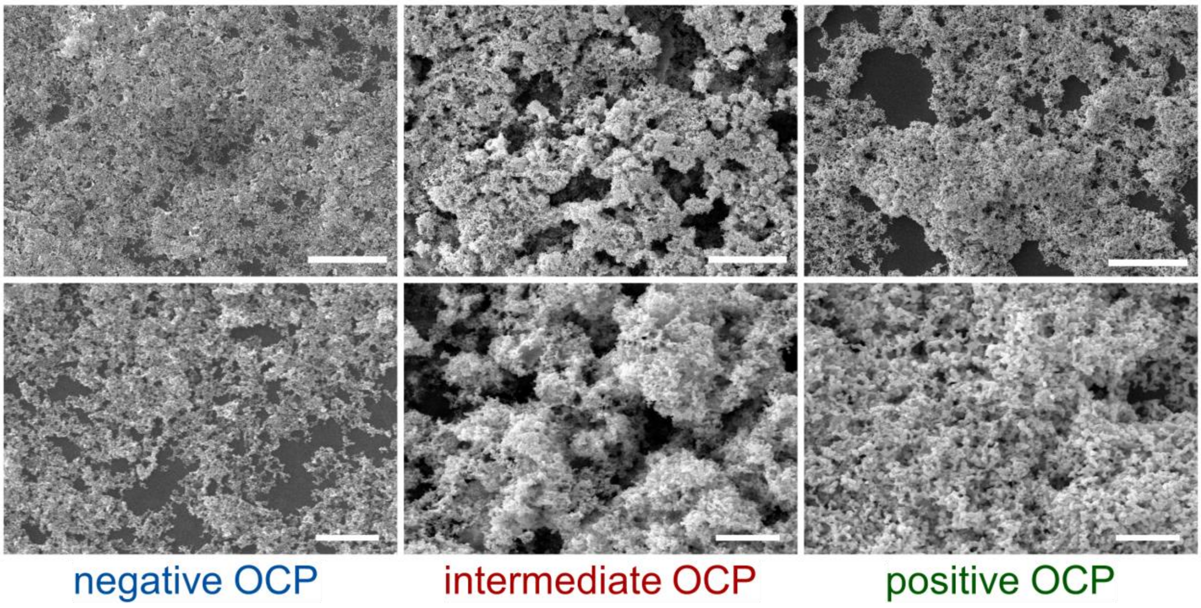

Fig. S9. SEM images of the morphologies of the AuNP films formed at an aqueous-TFT interface in the absence of 1 mM tolmetin in the aqueous phase. The aqueous-TFT interface was polarised at **(left column)** a negative OCP on the Galvani scale of –0.439 V using BACl electrolyte, **(middle column)** an intermediate OCP of -0.288 V using BATB electrolyte, and **(right column)** a positive OCP of +0.691 V using LiTB electrolyte. Top and bottom rows are SEM images of the same samples taken at different magnifications. Scale bars represent 5 µm (top row) and 1 µm (bottom row).

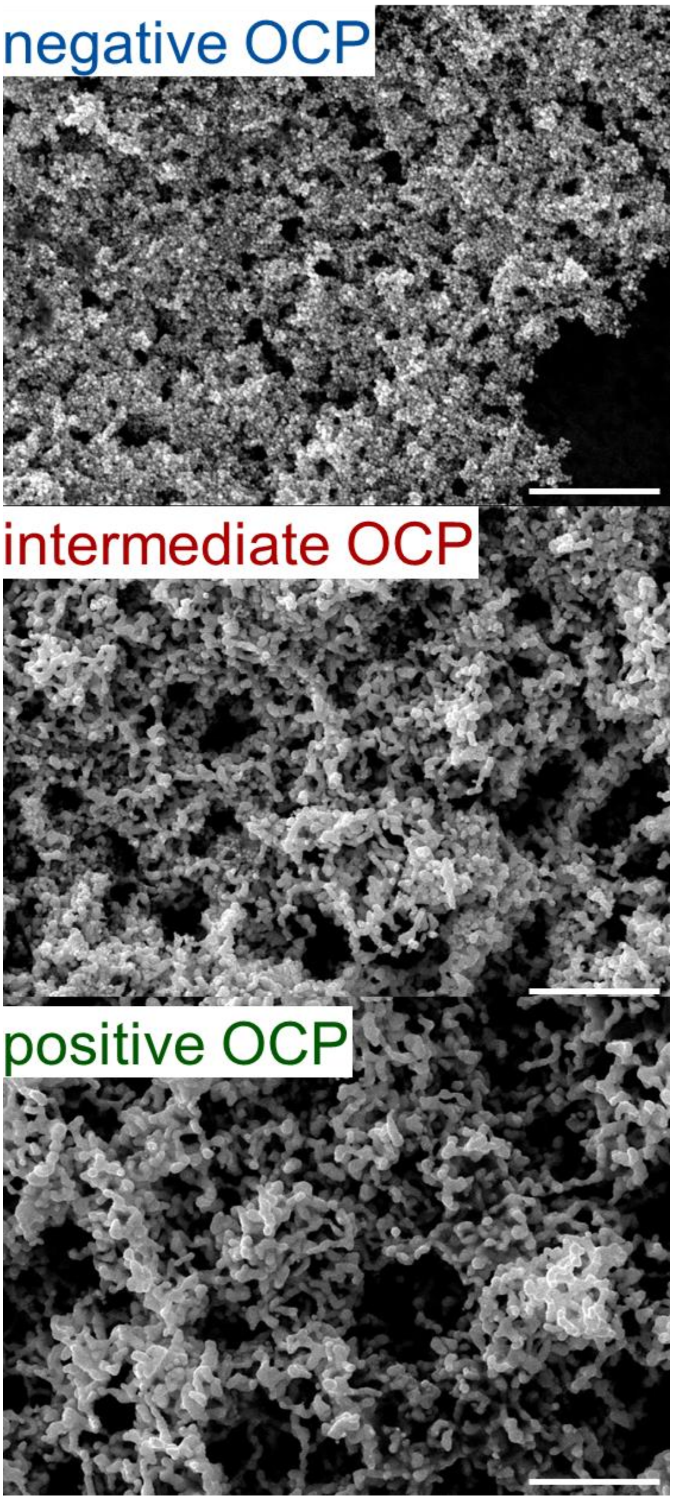

Fig. S10. SEM images of the morphologies of the AuNP films formed at an aqueous-TFT interface in the presence of 1 mM tolmetin in the aqueous phase. The aqueous-TFT interface was polarised at negative, intermediate, and positive OCPs on the Galvani scale as described in Fig. S9. The SEM images were obtained at a magnification of × 25,000 and the scalebar represents 1 µm.

S3.3 Fractal dimensions of the morphology of the interfacial AuNP films in the presence and absence of 1 mM tolmetin as a function of the polarisation of the aqueous-TFT interface

Fractal analyses were run on the SEM images obtained in the absence (see Fig. 2 (left column) of the main manuscript) and presence (see Fig. S10) of tolmetin. The calculations were based on the box-counting method.[7] The central idea of the method is to generate a grid defining squares that cover the image. By counting the number, *n*, of boxes that cover the black pixels as a function of the box length h, the fractal dimension D_f relates to the two quantities by the following equations:

$$n(h) \cong h^{-D_f} \qquad \text{Equation (S3)}$$

$$D_f = -\frac{\log(n)}{\log(h)} \qquad \text{Equation (S4)}$$

Therefore, one must define different box lengths h, count the associated number *n*, and plot -log(*n*) *versus* log(h). The points obtained are fitted by a linear function, the slope of the function giving D_f. ImageJ software uses box lengths of 2, 4, 8, 16, 32, 64, and 128 pixels.

The processing of the SEM images in the absence of tolmetin (Fig. S11) consisted of (i) applying a Sobel filter to the raw image to obtain the edges of the nanostructures, (ii) a binarisation, and (iii) the fractal analysis (plotted in Fig. S12). All the processing was performed using ImageJ software. D_f values are relative to the process used to treat the images, so their meaning is essentially relative rather than absolute. Since the process leads to binarised image, the scalebar was removed before analysis to avoid bias. An identical processing was carried out for the SEM images in the presence of tolmetin (data not shown). In the absence of tolmetin, D_f values of the films were 1.86, 1.80, and 1.76 for the negative, intermediate, and positive OCP, respectively. Meanwhile, in the presence of tolmetin, D_f values of the films were 1.96, 1.81, and 1.75 for the negative, intermediate, and positive OCP, respectively.

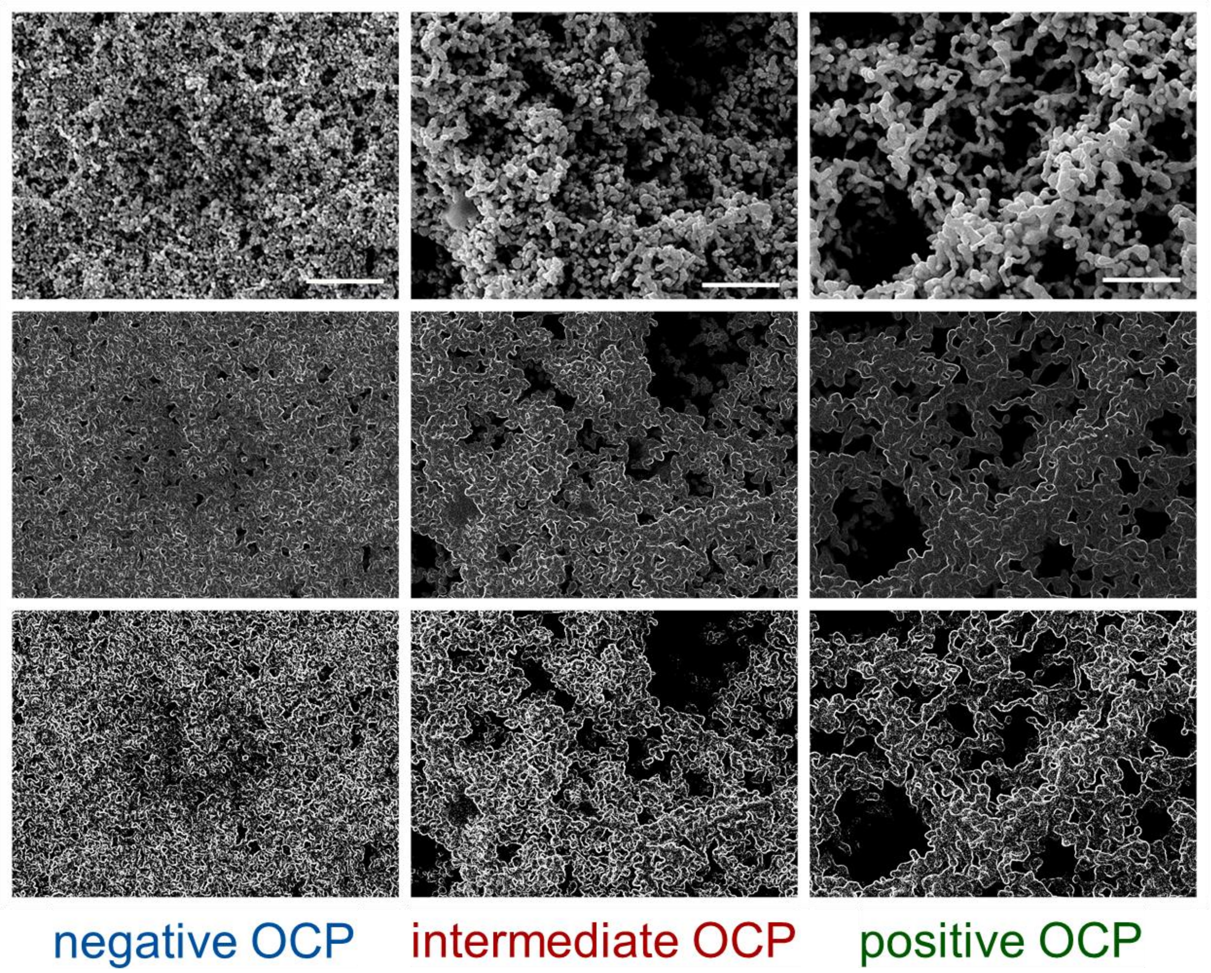

Fig. S11. (Top row) SEM images of the morphologies of the interfacial AuNP films formed in the absence of 1 mM tolmetin in the aqueous phase as a function of the polarisation of the aqueous-TFT interface. The scale bars represent 1 µm; **(middle row)** edges detection *via* Sobel filter of the SEM image using ImageJ software; **(bottom row)** binarisation of the images after Sobel filter.

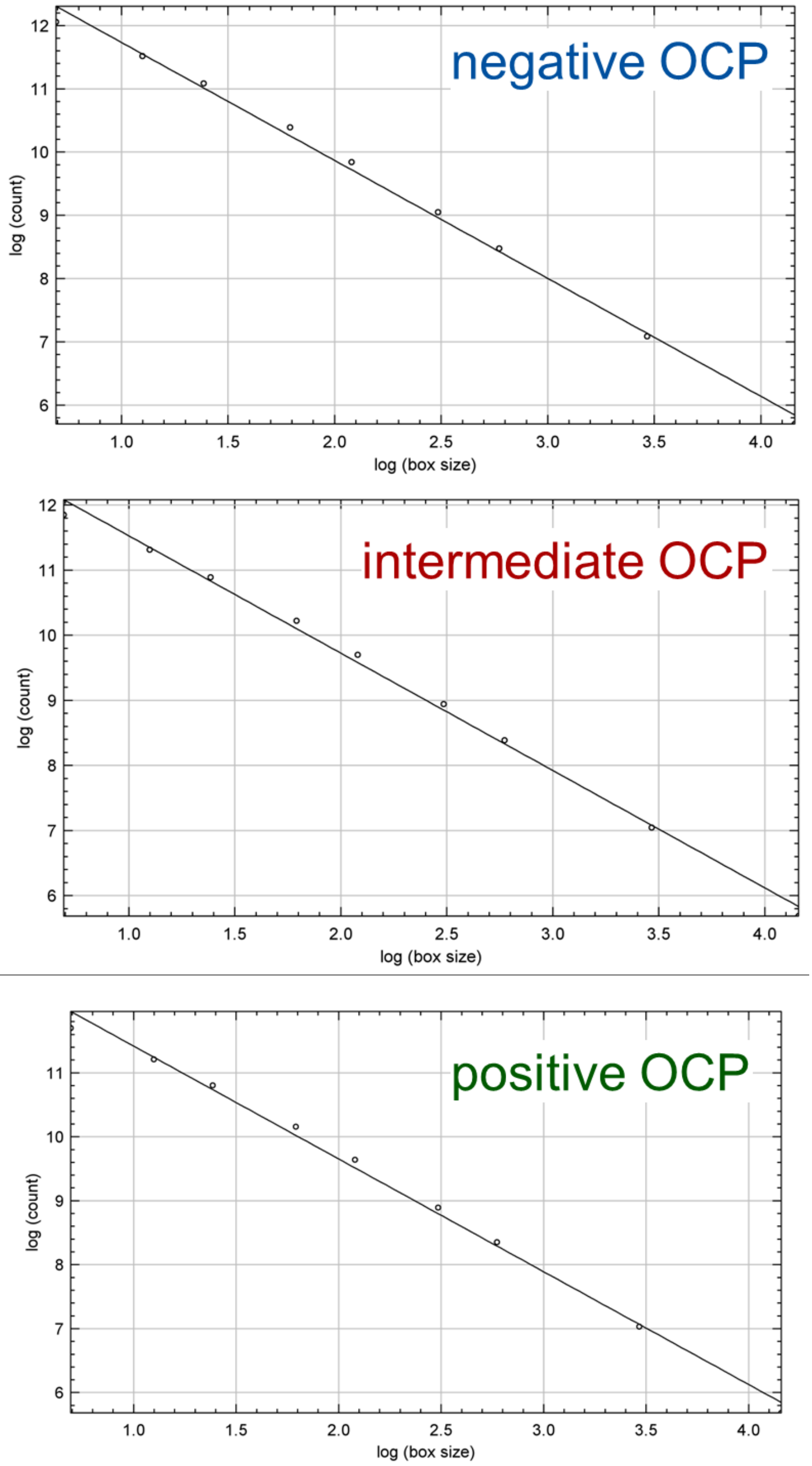

Fig. S12. Determination of the fractal dimension D_f using the $\log(h) = f(\log(n))$ plot for the interfacial AuNP films formed in the absence of 1 mM tolmetin in the aqueous phase as a function of the polarisation of the aqueous-TFT interface.

S4. Raman spectroscopy

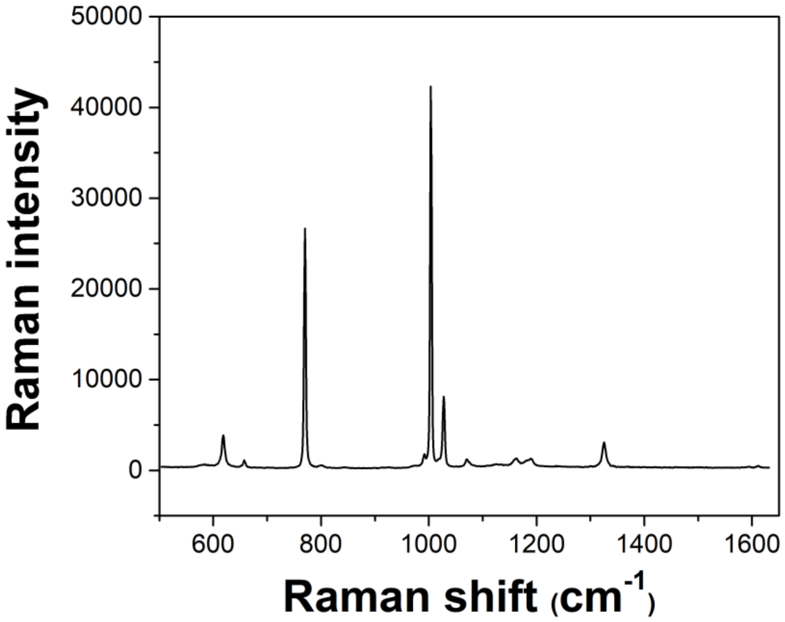

Fig. S13. Raman spectroscopy at a “bare” polarised interface formed between an aqueous solution of 100 mM NaCl and an organic solution containing 2.5 mM BATB in TFT solvent. This control experiment demonstrates the absence of intense Raman peaks of the aqueous and organic background electrolytes ions without the SERS effect from the interfacial AuNP film.

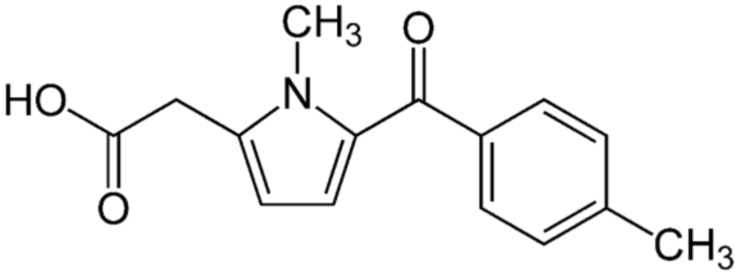

Fig. S14. Chemical structure of tolmetin.

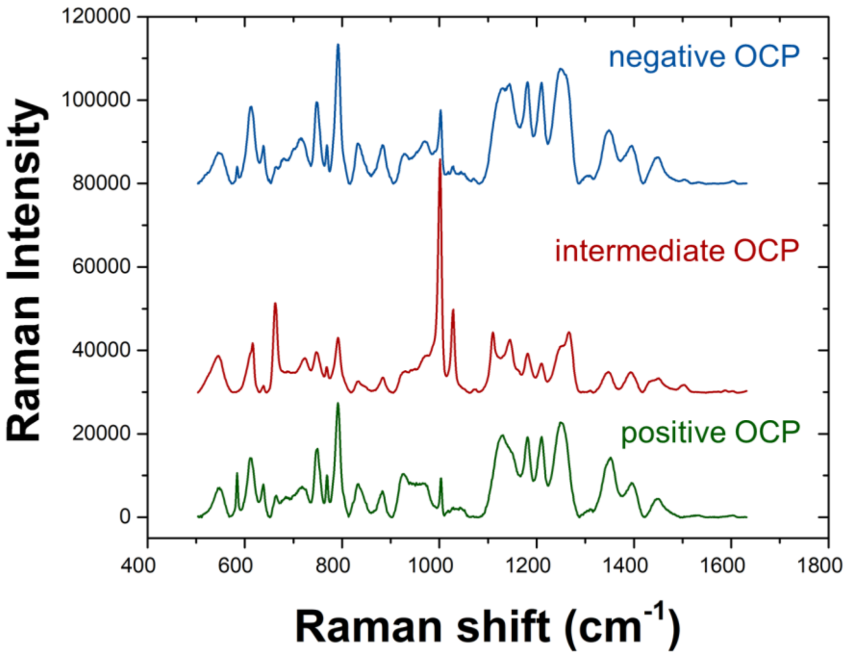

Fig. S15. SERS spectra of 1 mM tolmetin initially in the aqueous phase at interfacial AuNP films formed at negative, intermediate, and positive OCPs. All Raman spectra were acquired using a 785 nm laser at 1% power output.

To calculate the theoretical Raman spectrum of tolmetin, we performed modelling using Gaussian 09. Initially, after constructing the 3D molecule, we optimised it within the framework of molecular mechanics, using UFF (Universal Force Field). Once the molecule's energy reached its minimum, we conducted calculations within the realm of quantum mechanics to determine the vibrational frequencies. For this purpose, we employed DFT (Density Functional Theory) with a B3LYP/6-31G++(d,p) basis set. Upon achieving convergence in the calculations, we applied corrections related to the Raman vibrational modes using the equation established by Osaki *et al*:[8]

$$\bar{\sigma}_{scaled} = -0.0000104\bar{\sigma}_{calc}^2 + 0.9894\bar{\sigma}_{calc} \qquad \text{Equation (S5)}$$

Here, $\bar{\sigma}_{scaled}$ represents the corrected Raman shift in cm^{-1}, and $\bar{\sigma}_{calc}$ signifies the calculated DFT Raman shift. Fig. S16 showcases the excellent linear correlation discovered between the DFT-calculated, corrected frequencies, and the experimental values. In Table S1, we present the calculated and experimental frequencies, Raman band intensities, along with their assignments based on DFT calculations. Additionally, a supplementary column for peak

assignments sourced from the work of A. Jubert *et al* is included, serving as a basis for comparison.[9]

It is evident from the data that the theoretical spectrum aligns well with the experimental one for the frequencies. However, there is a noted discrepancy when it comes to the Raman intensities, a known aspect of DFT calculations.

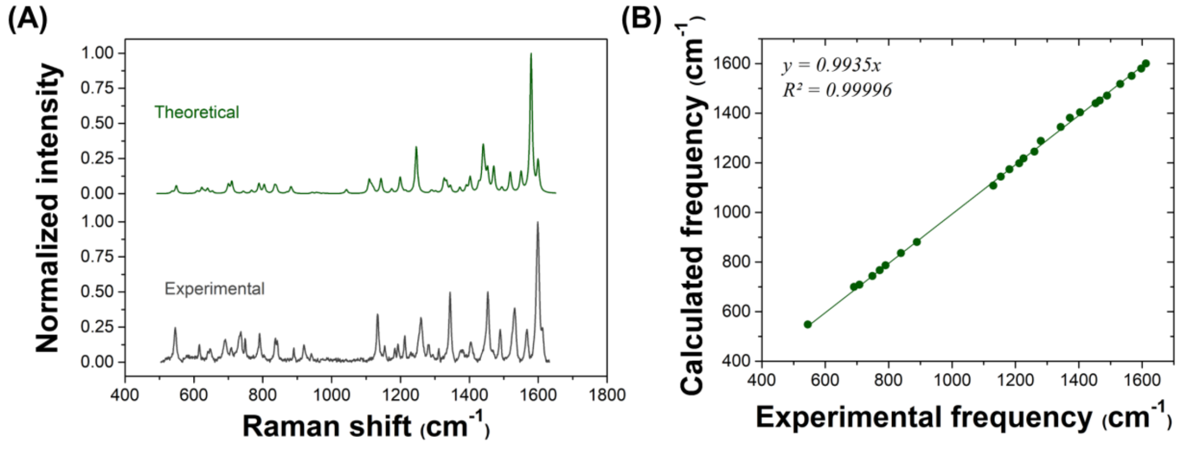

Fig. S16. (A) Experimental (bottom) and theoretical Raman spectra (top) for tolmetin. Experimental spectrum was obtained from a tolmetin solid grain (532 nm, 1%). **(B)** Correlation between the calculated frequencies and those experimentally obtained.

Table S1. Spectral assignment from DFT calculations of the most important vibrational peaks of Tolmetin between 500 and1650 cm^{-1}.

Raman shift / cm^{-1}		Normalised Raman Intensity		Assignment Exp	Assignment from A. Jubert et al.[9]
**Exp**	**Cal**	**Exp**	**Cal**		
**1612**	1600	0.25	0.25	- Aromatic C-C asymmetric stretching (s)<br>- ketone C=O stretching (w)	C-C ring stretching, in plane CH bending
**1597**	1580	1	1	- Aromatic C-C asymmetric stretching (w)<br>- ketone C=O stretching (s)	
**1567**	1550	0.23	0.16	- Aromatic C-C asymmetric stretching (w)<br>- ketone C=O stretching (w)	-Asymmetric N-Methyl def<br>- In plane Pyrrole ring def.<br>-C-C aromatic ring stretching
**1531**	1518	0.39	0.16	- Pyrrole C-C asymmetric stretching (s)	- In plane C-H aromatic ring bending<br>- C-C ring stretching
**1489**	1471	0.23	0.20	-N-Methylpyrrole C-H deformation (s)<br>- Pyrrole C-C asymmetric stretching (s)	- Pyrrole C-C stretching
**1466**	1451	0.09	0.19	- N-Methylpyrrole C-H deformation (s)<br>- Pyrrole C-C asymmetric stretching (s)	- Symmetric methyl deformation<br>- Pyrrole ring stretching<br>- C-N Pyrrole ring elongation<br>- In plane CH aromatic ring bending
**1453**	1440	0.5	0.35	Methyl groups C-H deformation (s)	

Raman shift / cm^{-1}		Normalised Raman Intensity		Assignment Exp	Assignment from A. Jubert et al.[9]
Exp	Cal	Exp	Cal		
**1404**	1403	0.14	0.14	- N-Methylpyrrole C-H out-of-plane deformation (s)<br>- Pyrrole C-C asymmetric stretching (m)	- Symmetric methyl deformation<br>- In plane Pyrrole ring deformation
**1380**	1372	0.09	0.04	- Aromatic methyl C-H out-of-plane deformation (s)	- Asymmetric methyl deformation
**1343**	1344	0.5	0.06	- Pyrrole C-C and C-N asymmetric stretching (s)	- C-C ring Pyrrole stretching<br>- In plane CH Pyrrole ring bending<br>- Symmetric methyl deformation<br>- In plane Pyrrole ring deformation
**1280**	1288	0.13	0.03	- C-H deformation (s)<br>- Aromatic C-C asymmetric stretching (w)<br>- Carboxylic C-O deformation	
**1260**	1245	0.32	0.34	- Pyrrole C-C asymmetric stretching (s) and C-H deformation	- C-C aromatic ring stretching<br>-in plane C-C aromatic ring deformation<br>- in plane CH ring bending<br>- In plane Pyrrole ring deformation
**1212**	1198	0.18	0.12	- Aromatic C-C breathing	- In plane CH ring bending
**1181**	1174	0.03	0.04	- Aromatic C-H deformation	- Methyl rocking
**1154**	1144	0.12	0.12	- Breathing of pyrrole and aromatic cycles	- In plane CH ring bending<br>- C-C aromatic ring stretching<br>- Pyrrole ring deformation

Raman shift / cm^{-1}		Normalised Raman Intensity		Assignment Exp	Assignment from A. Jubert et al.[9]
**Exp**	**Cal**	**Exp**	**Cal**		
**1130**	1108	0.35	0.11	- C-H bending	- In plan CH ring bending<br>- Carboxylic C-O-H deformation
**889**	881	0.11	0.05	- Aromatic C-C breathing<br>- Pyrrole C-C asymmetric stretching	- Out of plane CH ring bending<br>- In plane Pyrrole ring deformation
**839**	836	0.16	0.07	- Aromatic C-H bending	- Out of plane CH ring bending
**790**	787	0.21	0.09	- Aromatic C-C asymmetric stretching	- Out of plane CH ring bending.
**772**	767	0.06	0.03	- Aromatic and Pyrrole C-H bending	
**749**	744	0.18	0.03	- Pyrrole C-H bending	- Out of plane C-C ring bending<br>- Out of plane C-N Pyrrole ring bending
**708**	709	0.11	0.09	- C-C deformation	- Out of plane C-N Pyrrole ring bending.
**691**	700	0.17	0.08	- Aromatic C-C deformation	- Out of plane C-N Pyrrole ring deformation,
**545**	548	0.25	0.07	- C_{pyr}-C_{ketone}-$C_{aromatic}$ stretching	- O-H Elongation

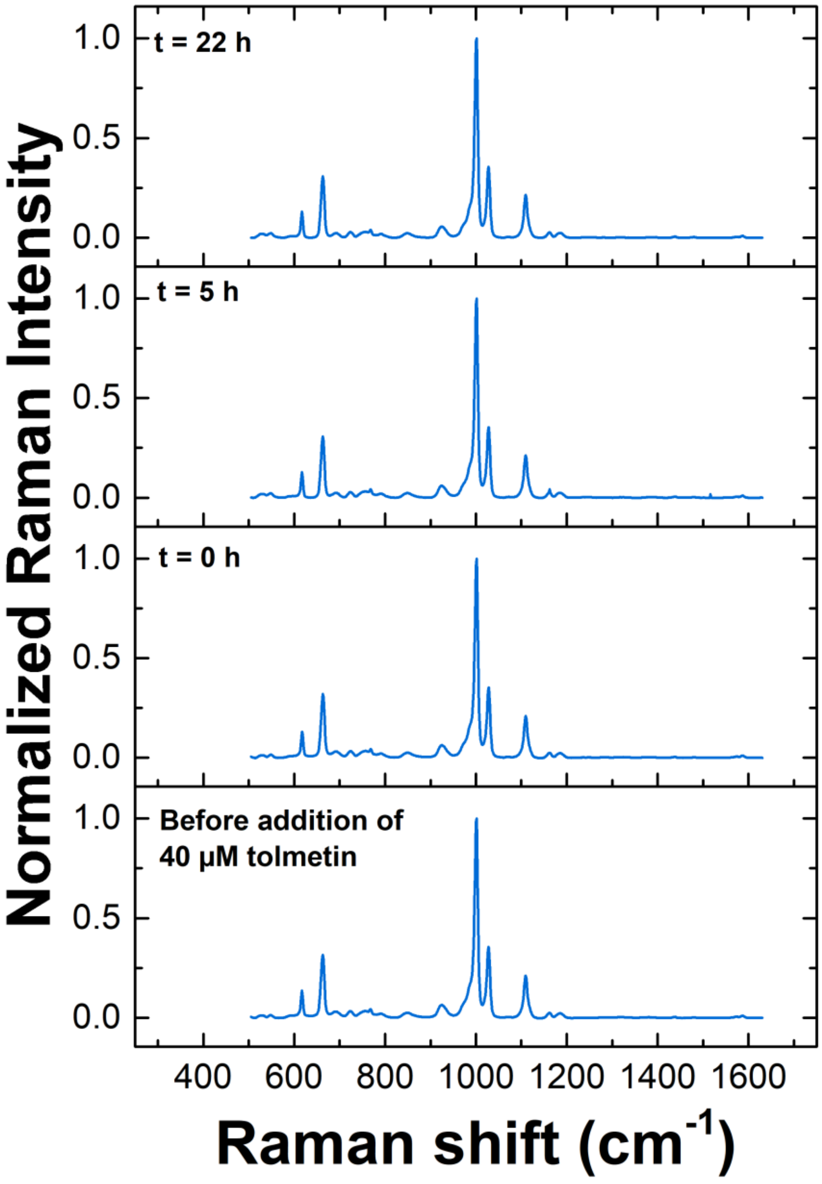

Fig. S17. SERS spectra at an interface modified with Au NP film before (bottom spectrum) and after the addition of 40 μM tolmetin to the aqueous phase. The AuNP film was formed under negative OCP. All Raman spectra were acquired straight after the addition of tolmetin (t = 0 h); after 5 h and after 22 h, using a 785 nm laser at 1% power output.

S5. Electrochemical behaviour of tolmetin at the polarisable aqueous-TFT interface

The electrochemical behaviour of the anionic form of tolmetin (p*K*a = 3.5) was investigated by CV and by AC voltammetry using the following electrochemical cell configuration:

Electrochemical cell 1: Ag/AgCl | 100 mM NaCl + *x* mM Tolmetin (pH 5.5) || TFT + 2.5 mM BATB |100 mM NaCl + 2.5 mM BACl | Ag/AgCl

In the absence of tolmetin (black curve, Fig. S18), the CV shows a current rise at the negative end of the polarisable potential window (PPW) corresponding to the transfer of hydrophilic Cl^- anions, and a current rise at positive end of the PPW corresponding to the transfer of hydrophilic Na^+ cations, from the aqueous to organic phase. Adding tolmetin to the aqueous phase led to an increase of the current observed at the negative end of the PPW. The background subtracted CV shows a weak current increase at -0.09 V, which could be attributed to the electrochemical behaviour of negatively charged tolmetin.

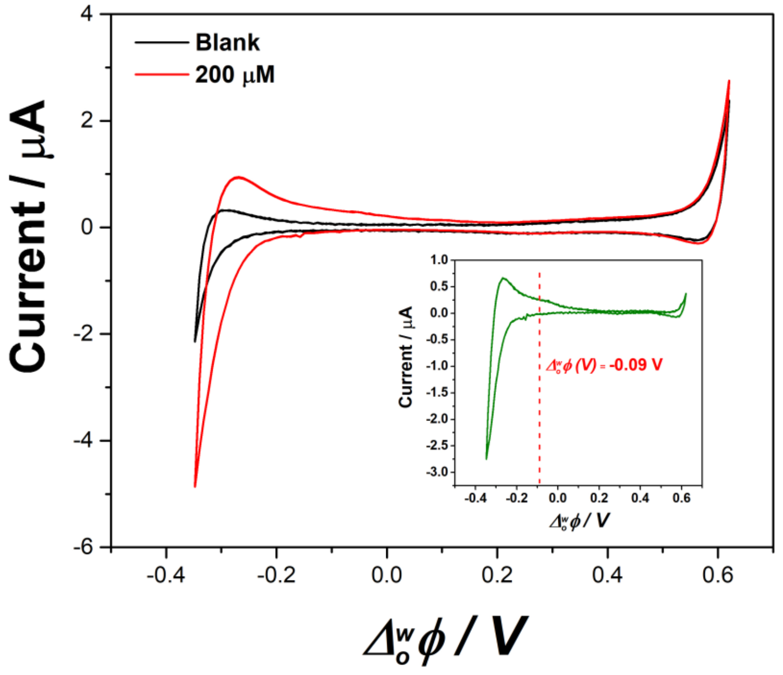

Fig. S18. CVs obtained in the absence (black line) and in the presence of 200 μM tolmetin (red). The scan rate was 5 mV·s^{-1} using electrochemical cell (1). Inset represents the CV after background subtraction.

S6. Determination of tolmetin biphasic distribution by UV-vis spectroscopy

S6.1 Single phase experiments: calibration curves of tolmetin in the aqueous and organic phases, respectively

Calibration curves for tolmetin were built in the aqueous phase (Fig. S20) and in the organic phase (Fig. S21). Unfortunately, it was not possible to build calibration curves in the presence of the organic electrolyte anion TB^- as there was an overlap of absorbance.

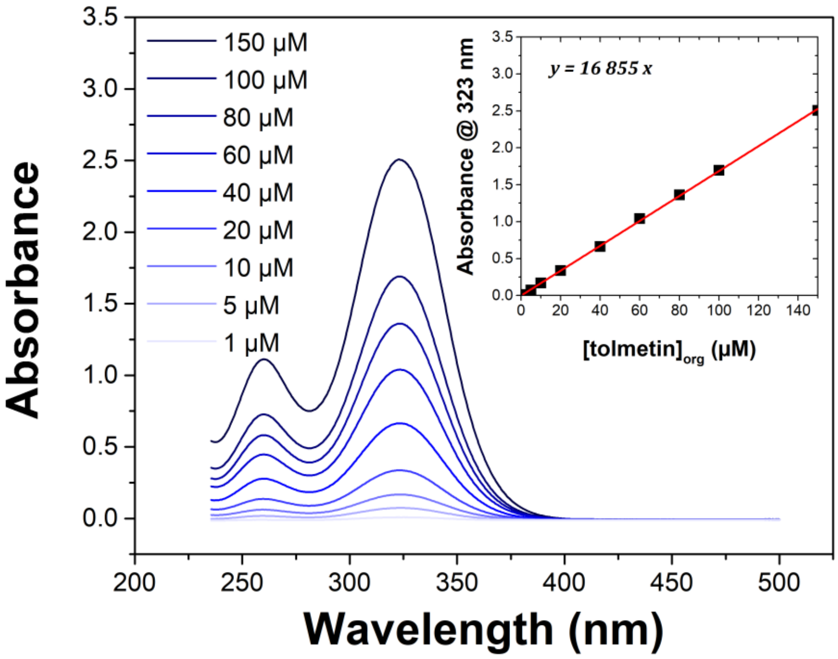

Fig. S19. UV-vis absorption spectra of 1, 5, 10, 20, 40 ,60 ,80 ,100, and 150 µM tolmetin in 100 mM NaCl. Inset: calibration curve for tolmetin based on the absorbance at 323 nm.

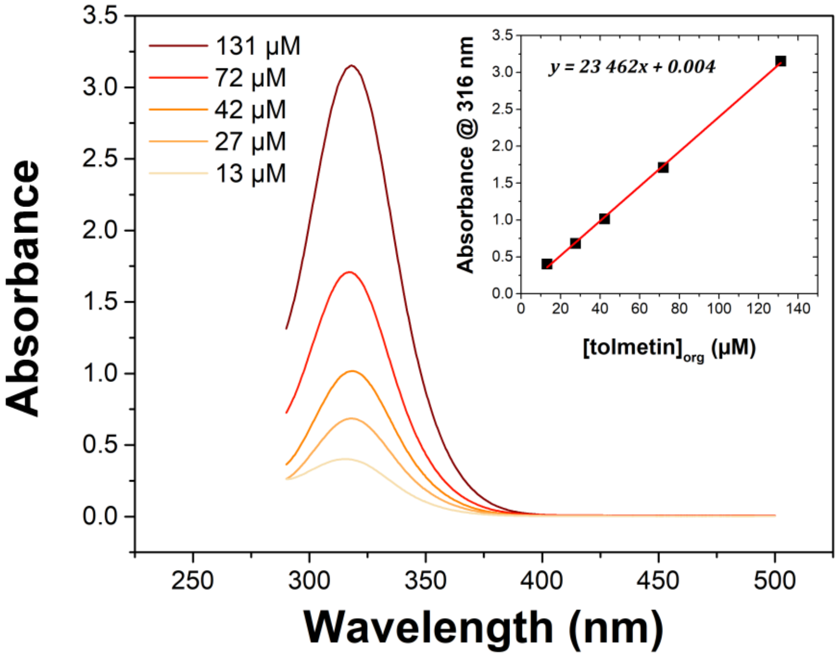

Fig. S20. UV-vis absorption spectra of 13, 27, 42, 72, and 131 μM tolmetin in 1 mM BACl in TFT. Inset: calibration curve for tolmetin based on the absorbance at 316 nm.

S6.2 Biphasic experiments: UV-vis absorption spectra of tolmetin in the aqueous and organic phases, respectively, at equilibrium

In this series of experiments, the aim was to determine the distribution of tolmetin molecules between the aqueous phase, the interfacial AuNP film, and the organic phase. An interfacial AuNP film was formed in the presence of various concentrations of tolmetin (ranging from 1.6 μM to 1 mM) and the cell was let to settle for 5 h. After this time, tolmetin concentrations were determined by UV-vis absorption spectroscopy in both the aqueous (Fig. S21A) and organic (Fig. S21B) phases.

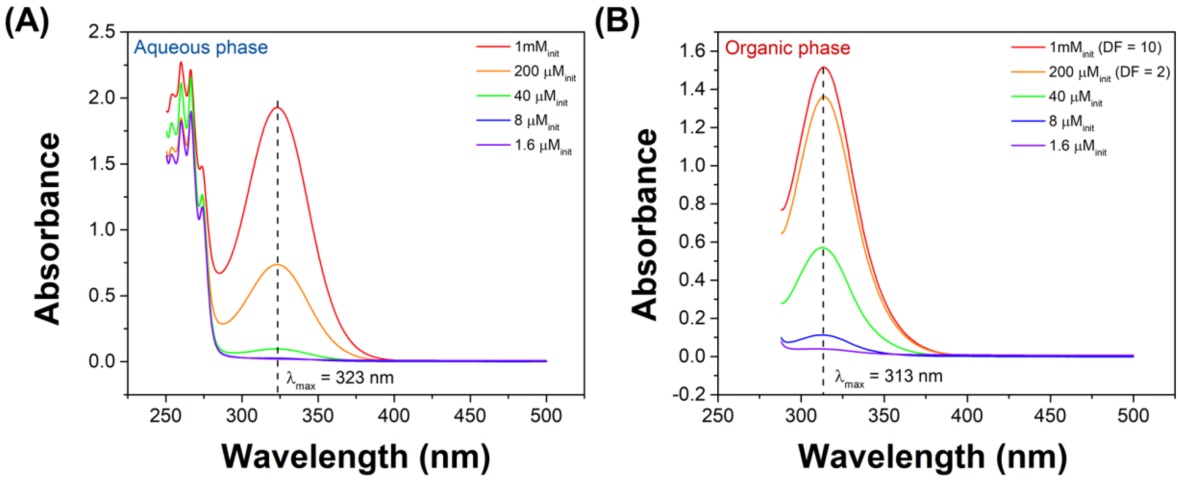

Fig. S21. Determination of the tolmetin concentration by UV-vis absorption spectroscopy in **(A)** the aqueous phase and **(B)** the organic phase after an interfacial AuNP film was formed at a negative OCP set by BACl. DF stands for the dilution factor needed to stay within the concentration range of the calibration curve built in Fig. S20. Both phases were collected after 5 h.

The concentration of tolmetin molecules that remain in the aqueous phase and the concentration of tolmetin molecules that have transferred to the organic phase are then calculated. The amount of tolmetin molecules adsorbed onto the Au NP film and the number of molecules per nm^2, θ, are then deduced using Equations S6 and S7.

$$n_{tolmetin}^{film} = n_{tolmetin}^{total} - \left(n_{tolmetin}^{aqueous} + n_{tolmetin}^{organic}\right) \qquad \text{Equation (S6)}$$

$$\theta = \frac{n_{tolmetin}^{film} N_A}{N\pi d^2} \qquad \text{Equation (S7)}$$

where N_A is Avogadro's number, N is the number of Au NPs as defined by Equation (S2) and d the nanoparticle diameter calculated using Equation (S1).

Table S2. Estimation of the proportion of the initial concentration of tolmetin in the aqueous phase that remains in the aqueous phase, $\frac{n_{tolmetin}^{aq}}{n_{tolmetin}^{total}}$, transfers to the organic phase, $\frac{n_{tolmetin}^{org}}{n_{tolmetin}^{total}}$, or is adsorbed onto the interfacial AuNP film, $\frac{n_{tolmetin}^{film}}{n_{tolmetin}^{total}}$. From this data (in conjunction with other data as described in the main text), the number of tolmetin adsorbed per surface area of gold (molecules/nm^2) was calculated. These results were obtained for a negative OCP set by BACl.

**[tolmetin] / µM**	$\frac{n_{tolmetin}^{aq}}{n_{tolmetin}^{total}}$	$\frac{n_{tolmetin}^{org}}{n_{tolmetin}^{total}}$	$\frac{n_{tolmetin}^{film}}{n_{tolmetin}^{total}}$	θ **Molecules/nm^2**
1000	0.12	0.64	0.24	264
200	0.22	0.58	0.20	43
40	0.15	0.61	0.24	3
8	0.20	0.58	0.22	0.5

When the initial tolmetin concentration is 1.6 µM, UV-vis absorption spectroscopy is not sufficiently sensitive to quantify the concentration of tolmetin in either aqueous or organic phase.

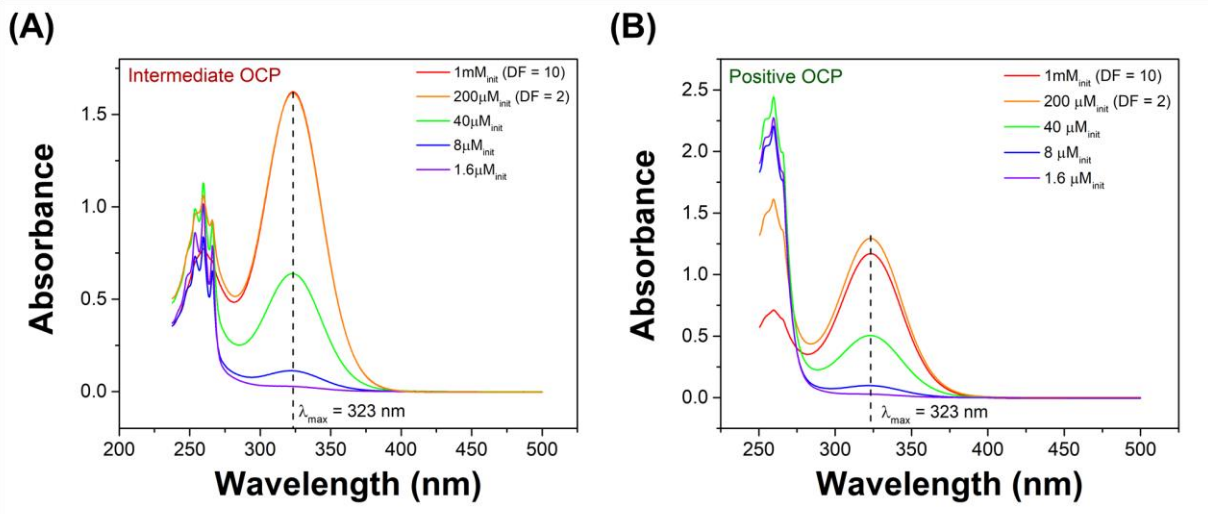

Fig. S22. Determination of the tolmetin concentration by UV-vis absorption spectroscopy in the aqueous phase at equilibrium when the interfacial polarisation was set to **(A)** the intermediate OCP by BATB and **(B)** the positive OCP by LiTB. The aqueous phases in each case were collected after 5 h. DF stands for the dilution factor needed to stay within the concentration range of the calibration curve built in Fig. S19.

Table S3. Tolmetin concentrations remaining in the aqueous phase for various initial tolmetin concentrations. These values were obtained for an intermediate OCP set by BATB and a positive OCP set by LiTB.

	$[\boldsymbol{tolmetin}]_{\boldsymbol{aq}}^{\boldsymbol{initial}}$ **μM**	$[\boldsymbol{tolmetin}]_{\boldsymbol{aq}}^{\boldsymbol{equiibrium}}$ **μM**	**% remaining in aqueous phase**
Intermediate OCP	1000	961.1	96.1
	200	191.0	95.5
	40	37.9	94.7
	8	6.6	82.5
Positive OCP	1000	693.9	69.4
	200	153.5	76.7
	40	30.0	75.0
	8	5.8	72.5

S7. Supplementary references

(1) Smirnov, E.; Peljo, P.; Scanlon, M. D.; Girault, H. H. Gold Nanofilm Redox Catalysis for Oxygen Reduction at Soft Interfaces. *Electrochim. Acta* **2016**, *197*, 362–373. https://doi.org/10.1016/j.electacta.2015.10.104.

(2) Kimling, J.; Maier, M.; Okenve, B.; Kotaidis, V.; Ballot, H.; Plech, A. Turkevich Method for Gold Nanoparticle Synthesis Revisited. *J. Phys. Chem. B* **2006**, *110* (32), 15700–15707. https://doi.org/10.1021/jp061667w.

(3) Haiss, W.; Thanh, N. T. K.; Aveyard, J.; Fernig, D. G. Determination of Size and Concentration of Gold Nanoparticles from UV−Vis Spectra. *Anal. Chem.* **2007**, *79* (11), 4215–4221. https://doi.org/10.1021/ac0702084.

(4) Hung, L. Q. Electrochemical Properties of the Interface between Two Immiscible Electrolyte Solutions. Part I. Equilibrium Situation and Galvani Potential Difference. *J. Electroanal. Chem.* **1980**, *115* (2), 159–174. https://doi.org/10.1016/S0022-0728(80)80323-8.

(5) Iwata, T.; Nagatani, H.; Osakai, T. Determination of the Electrostatic Potential of Oil-in-Water Emulsion Droplets by Combined Use of Two Membrane Potential-Sensitive Dyes. *Anal. Sci.* **2017**, *33* (7), 813–819. https://doi.org/10.2116/analsci.33.813.

(6) Trojánek, A.; Mareček, V.; Langmaier, J.; Samec, Z. Effect of Water Solubility in Organic Solvents on the Standard Gibbs Energy of Ion Transfer across a Water/Organic Solvent Interface. *Electrochim. Acta* **2023**, *449* (January), 142222. https://doi.org/10.1016/j.electacta.2023.142222.

(7) Abdellatif, M. H.; Abdelrasoul, G. N.; Salerno, M.; Liakos, I.; Scarpellini, A.; Marras, S.; Diaspro, A. Fractal Analysis of Inter-Particle Interaction Forces in Gold Nanoparticle Aggregates. *Colloids Surfaces A Physicochem. Eng. Asp.* **2016**, *497*, 225–232. https://doi.org/10.1016/j.colsurfa.2016.03.013.

(8) Osaki, T.; Soejima, E. Quadratic Scaling Functions for Obtaining Normal Vibrational Wavenumbers from the B3LYP Calculation. *Res. Bull. Fukuoka Inst. Tech.* **2010**, *2*, 129–134.

(9) Jubert, A.; Legarto, M. L.; Massa, N. E.; Tévez, L. L.; Okulik, N. B. Vibrational and Theoretical Studies of Non-Steroidal Anti-Inflammatory Drugs Ibuprofen [2-(4-Isobutylphenyl)Propionic Acid]; Naproxen [6-Methoxy-α-Methyl-2-Naphthalene

Acetic Acid] and Tolmetin Acids [1-Methyl-5-(4-Methylbenzoyl)-1H-Pyrrole-2-Acetic Acid]. *J. Mol. Struct.* **2006**, *783* (1–3), 34–51. https://doi.org/10.1016/j.molstruc.2005.08.018.
